# Males That Silence Their Father’s Genes: Genomic Imprinting of a Complete Haploid Genome

**DOI:** 10.1093/molbev/msab052

**Published:** 2021-03-04

**Authors:** Andrés G de la Filia, Andrew J Mongue, Jennifer Dorrens, Hannah Lemon, Dominik R Laetsch, Laura Ross

**Affiliations:** School of Biological Sciences, Institute of Evolutionary Biology, The University of Edinburgh, Edinburgh, United Kingdom

**Keywords:** paternal genome elimination, genomic imprinting, genomic conflict, allele-specific expression, mealybugs

## Abstract

Genetic conflict is considered a key driver in the evolution of reproductive systems with non-Mendelian inheritance, where parents do not contribute equally to the genetic makeup of their offspring. One of the most extraordinary examples of non-Mendelian inheritance is paternal genome elimination (PGE), a form of haplodiploidy which has evolved repeatedly across arthropods. Under PGE, males are diploid but only transmit maternally inherited chromosomes, while the paternally inherited homologues are excluded from sperm. This asymmetric inheritance is thought to have evolved through an evolutionary arms race between the paternal and maternal genomes over transmission to future generations. In several PGE clades, such as the mealybugs (Hemiptera: Pseudococcidae), paternal chromosomes are not only eliminated from sperm, but also heterochromatinized early in development and thought to remain inactive, which could result from genetic conflict between parental genomes. Here, we present a parent-of-origin allele-specific transcriptome analysis in male mealybugs showing that expression is globally biased toward the maternal genome. However, up to 70% of somatically expressed genes are to some degree paternally expressed, while paternal genome expression is much more restricted in the male reproductive tract, with only 20% of genes showing paternal contribution. We also show that parent-of-origin-specific gene expression patterns are remarkably similar across genotypes, and that genes with completely biparental expression show elevated rates of molecular evolution. Our results provide the clearest example yet of genome-wide genomic imprinting in insects and enhance our understanding of PGE, which will aid future empirical tests of evolutionary theory regarding the origin of this unusual reproductive strategy.

## Introduction

Sex—the mixing of heritable material of two individuals—is nearly universal among multicellular organisms. Yet the variety of ways organisms achieve this is staggering. For example, organisms can differ in the way genetic material is inherited, how the genes of two parents are combined to form offspring, or how the sex of the offspring is determined. Why there is such large variability in a process that is so fundamental remains one of the unsolved mysteries of life. A rapidly growing body of theoretical literature suggests that conflicts between the sexes play a central role in driving the rapid turnover of reproductive and sex determining systems (Hurst et al. 1996; van Doorn and Kirkpatrick 2007; van Doorn and Kirkpatrick 2010; Bachtrog et al. 2014; Gardner and Ross 2014; Úbeda et al. 2015). These conflicts are particularly pronounced during reproduction, as they force unrelated mates to cooperate, and whilst this cooperation is aimed at achieving a common goal—producing descendants—not all interests are aligned (Chapman et al. 2003).

One particular conflict between parents that could have led to the evolution of new reproductive strategies is over inheritance of their respective genes by future generations (intragenomic conflict between parents: Hurst 1992; Haig 2000; Burt and Trivers 2006; Ross et al. 2010; Werren 2011; Normark and Ross 2014; Gardner and Úbeda 2017). Despite these conflicts, in many species—like ourselves—reproduction is a fair process that follows Mendel’s classic law of inheritance: each parent contributes an equal share of the heritable material of their offspring (Wright 1931). Yet in some species (∼15% of animals), rules of fair reproduction are violated (Bachtrog et al. 2014). Probably the best-known example is haplodiploidy—found in wasps, ants and bees—where sons only inherit their mother’s and not their father’s genes, but many other examples are found across the animal kingdom. Extensive theoretical work has argued that such non-Mendelian reproduction could evolve as a result of conflict between the sexes, but empirical tests are sorely needed to test whether the plausible is actual.

To date, most empirical work exploring this conflict hypothesis has focused on mammals and other model organisms. Yet one of the most extreme and widespread cases of non-Mendelian inheritance has barely been explored: in species with paternal genome elimination (PGE), males develop from fertilized eggs, but the complete haploid set of chromosomes inherited from their fathers is eliminated at some stage and excluded from sperm (Normark 2003; Burt and Trivers 2006; Gardner and Ross 2014; Blackmon et al. 2015). PGE has evolved independently at least seven times across arthropods and is estimated to be present in >10,000 species (de la Filia et al. 2015). Under PGE, both sexes are diploid, in contrast to classic haplodiploidy—but, as in the latter, males only transmit maternally inherited chromosomes to their offspring. Therefore, transmission of genes is not random in males, but dependent on whether they are maternally derived. This provides a transmission advantage to mothers through their sons, and the evolution of PGE has been frequently framed as an outcome of intragenomic conflict between parental genomes in males—and therefore between the males’ parents—by maternally inherited genomes being able to manipulate spermatogenesis to enhance their own transmission (Bull 1979; Herrick and Seger 1999; Ross et al. 2010; Normark and Ross 2014; Anderson et al. 2020). However, even if parental conflict was responsible for the initial evolution of PGE, it is unclear if this conflict is ongoing in extant species.

In addition to biased gene transmission, PGE can also affect the chromatin state of paternal chromosomes in males, which has been suggested to result in extreme parent-of-origin-dependent gene expression (genomic imprinting). In several species where elimination of paternal chromosomes is postponed until spermatogenesis (germline PGE)—such as mealybugs, coffee borer beetles, and the booklice *Liposcelis*—paternal chromosomes are heavily condensed and compacted into a dense body at the periphery of the nucleus, reminiscent of the mammalian bar body (Borgia 1980; Nur 1990; Hodson et al. 2017). This heterochromatinization process takes place during embryogenesis, and, as a result, males—despite being genetically diploid—are thought to become functionally haploid, only expressing maternal alleles (Prantera and Bongiorni 2012). Like other cases of parent-of-origin specific gene expression, such as genomic imprinting of single genes in mammals and flowering plants (Reik and Walter 2001; Ferguson-Smith 2011), this is thought to result from intragenomic conflict between maternally and paternally derived alleles: silencing of the paternal genome in males could prevent it from expressing anti-PGE adaptations to escape elimination and restoring fair Mendelian transmission or from reducing male fitness under sibling competition (Herrick and Seger 1999; Ross et al. 2010; Ross et al. 2011). Although paternal genome silencing has evolved repeatedly in the context of PGE, the hypothesis that it has evolved as an outcome of genomic conflict remains to be tested and, crucially, a more complete understanding of paternal genome silencing in PGE males is still lacking.

In order to address this, we study paternal genome silencing in mealybugs (Hemiptera; Pseudococcidae), small plant-feeding insects that reproduce through PGE. As in all germline PGE taxa, heterochromatization of the paternal genome only occurs in males, while females do not exhibit this phenomenon and are normally diploid. Therefore, we present a RNA-seq allele-specific expression analysis (ASE) (Wang and Clark 2014) in male offspring of hybrid and intraspecific crosses, which allowed us to detect and quantify parent-of-origin effects on gene expression at a whole-genome scale in the sex directly affected by PGE.

We focus on three key questions: first of all, is the paternal genome completely inert, or do some paternal genes escape silencing? Earlier work suggesting the completely silenced state of the paternal genome relied on cytogenetic observations or expression of a small number of phenotypic traits or genetic markers (Brown and Nur 1964; Brown and Wiegmann 1969; Brown 1972; Brun et al. 1995; Borsa and Kjellberg 1996), and therefore lack the genome-wide resolution to address this question. Complete silencing of paternally inherited chromosomes would indicate that there is very little scope for ongoing intragenomic conflict within mealybug males; however, any genes that escape silencing might be involved in conflict (Herrick and Seger 1999; Ross et al. 2010; Ross et al. 2011). Second, does the extent of paternal genome silencing differ between somatic and reproductive tissues? Previous studies in *Drosophila*, a diplodiploid species, show that sexually antagonistic loci are underrepresented in the testis, compared with somatic tissues (Innocenti and Marrow 2010). However, the unique selective pressures on the parental genome under PGE create scope for intragenomic conflict in reproductive tissues: paternal chromosomes are eliminated during spermatogenesis, so we might expect the reproductive tract (both the germline and the somatic parts of testes) to be a hotspot for intragenomic conflict and experience stronger selection for silencing of the paternal genome. We test this hypothesis by comparing patterns of parent-of-origin expression between male soma and reproductive tract. Finally, do biparentally expressed genes evolve faster than genes with complete maternal expression? Evolutionary conflict can lead to arms races in which each party evolves rapidly in response to the harm inflicted by the other. As a result, genes involved in this conflict might evolve rapidly (Geist et al. 2019). We therefore test if genes that escape paternal genome silencing, particularly those in reproductive tissues, show an elevated rate of molecular evolution.

We find clear evidence that expression in males is heavily, but not completely, biased toward the maternal genome, both in hybrids and pure *Planococcus citri* males, indicating consistent genome-wide imprinting in PGE males which is particularly strong in the reproductive tract. However, a fraction of genes show transcriptional contribution of paternal chromosomes and a few are completely biparental, especially in somatic tissues, and we show that biparentally expressed genes evolve at an accelerated rate compared with completely maternally expressed genes. The different extent of paternal genome silencing between soma and reproductive tract and the difference in rates of evolution between maternally and biparentally expressed genes are consistent with the intragenomic conflict hypothesis, although other alternative explanations cannot be ruled out.

## Results

In order to determine whether paternal genomes retain transcriptional activity or are completely silenced in male mealybugs (supplementary fig. S1, Supplementary Material online), we estimated patterns of ASE in 1) somatic and reproductive tissues of adult hybrid males (CF males) originated in crosses between citrus mealybug (*P. citri*) females and males from the closely related vine mealybug (*Planococcus ficus*), and 2) whole adult *P. citri* males produced in reciprocal intraspecific crosses between three pairs of isofemale lines (WYE3-2 × CP1-2, WYE3-2 × BGOX-6, and CP1-2 × BGOX-6). A detailed overview of experimental methods and analysis strategy is given in Materials and Methods.

### ASE in Hybrid Males

**Fig. 1.**
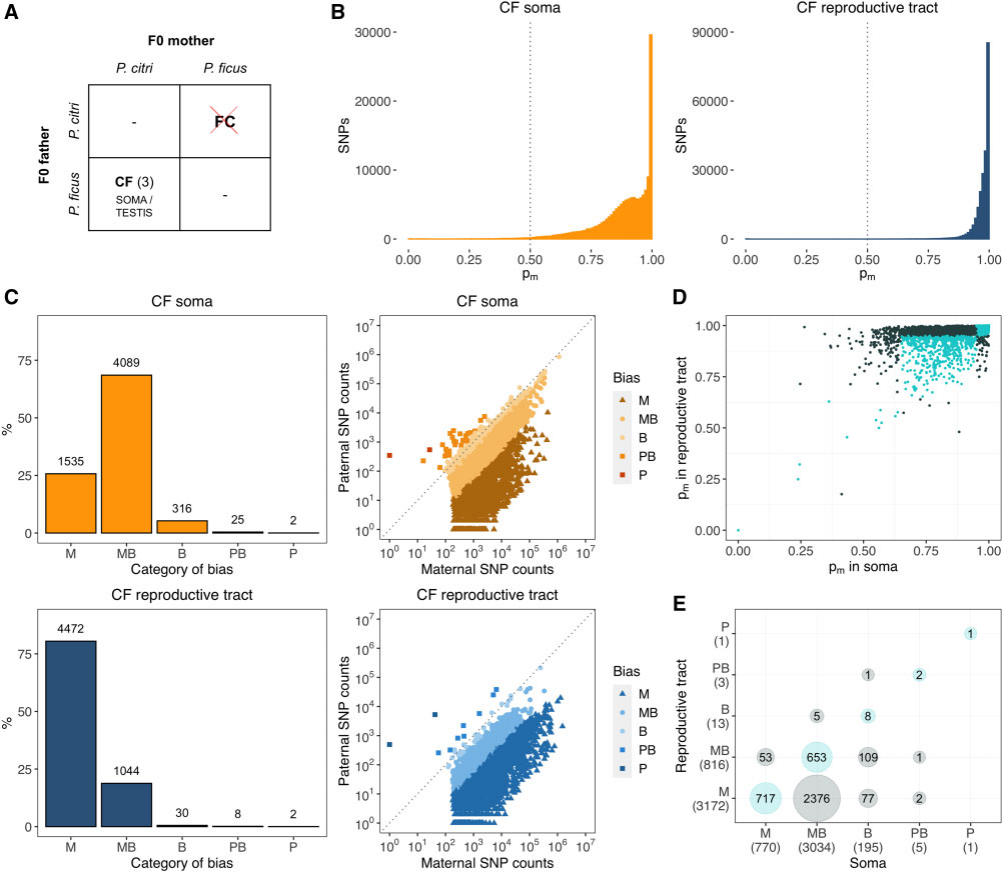
Quantification of allele-specific expression (ASE) in soma and reproductive tract of hybrid F1 mealybug males. (*A*) Cross scheme between *Planococcus citri* and *Planococcus ficus.* Only CF crosses produced viable adult male offspring (number of replicates in brackets). Reproductive tracts from CF males were dissected and sequenced separately from the soma. (*B*) Histogram of expression biases to maternal genome, *p_m_*, at SNP level in soma (orange, left) and reproductive tract (blue, right) of F1 mealybug males (pooled maternal and paternal counts between replicates). The dotted line indicates complete biparental expression. (*C*) Counts of genes with allele-specific information according to category of ASE expression (from completely maternal, M, to completely paternal, P) (left panels) and scatterplots of combined paternal and maternal counts (across exonic SNPs and replicates) for genes expressed in soma and reproductive tract (right panels). (*D*) Scatterplot of *p_m_* in soma and reproductive tract for all genes with allele-specific information expressed in both tissue types (genes belonging to the same ASE category in both are shown in turquoise). (*E*) Cross table of overlapping gene counts by ASE category in soma and reproductive tract.

We obtained transcriptomes from three biological replicates of somatic tissues and the reproductive tract dissected from pools of CF hybrid males (113–158 M reads per sample) (fig. 1*A*). Unfortunately, we could not raise viable adult male offspring of the reciprocal FC cross (*P. ficus* females × *P. citri* males), as these F1 males die in early larval stages, and therefore we were unable to obtain transcriptomes from the reciprocal cross.

We aligned the RNA-seq reads to a custom pseudogenome constructed from the PCITRI.V0 reference genome (https://ensembl.mealybug.org/, last accessed March 02, 2021) and a panel of 4,670,197 interspecific SNPs between the parental *P. citri* and *P. ficus* genomes (Materials and Methods). We found and validated 159,059 and 221,600 SNPs from the interspecific panel in all three CF soma and reproductive tract transcriptomes, respectively, at a read depth of at least 30× (supplementary fig. S2, Supplementary Material online). At each SNP, we estimated the proportion of reads that originated from the maternal genome (*p_m_*) and found that the vast majority of reads are of maternal origin in both soma (0.88 ± 0.13) and reproductive tract (0.97 ± 0.06) (fig. 1*B*, supplementary table S1, Supplementary Material online), as expected under heterochromatinization of paternal chromosomes.

To estimate ASE at gene level, we assigned to coding regions 93,039 SNPs in the soma (59% of total) and 101,333 in the reproductive tract (46%). Out of 18,667 genes with detectable expression levels (TPM > 1) in soma and 15,286 in the reproductive tract, we were able to confidently estimate ASE in 5,967 and 5,556 genes, respectively. These genes are those covered by at least two SNPs in all three tissue replicates (or a single SNP with average read depth > 100), and showing homogeneity in expression bias across tissue replicates (Materials and Methods). To generate gene-level ASE estimates for each of these genes, we pooled maternal and paternal read counts across all exonic SNPs (on average, 14.7 SNPs per gene) in each individual replicate and estimated genic *p_m_* as the fraction of reads originated from the maternal genome. Since both somatic and reproductive tissue replicates show high consistency in genic *p_m_* estimations (average *p_m_* difference between replicates of |0.026| and |0.017|, respectively), we obtained a single *p_m_* estimate for each gene in soma and reproductive tract by pooling maternal and paternal counts from all three replicates.

Concordantly with the patterns observed at SNP level, gene expression is globally biased toward the maternal genome in both somatic and reproductive tissues (fig. 1*C*). In soma, only 5.2% of genes (316) exhibit biparental (B) expression, defined (after Wang and Clark 2014) as *p_m_* = 0.35–0.65 and/or Bonferroni-corrected exact binomial test versus *p_m_* = 0.5 not rejected. The majority of somatic genes (68.5%, 4,089 genes) are predominantly expressed from the maternal *P. citri* genome (maternally biased, MB, with 0.65 < *p_m_* < 0.95) and a further 25.7% of genes (1,532) are completely maternal (M, *p_m_* ≥ 0.95). Additionally, we found 25 somatic genes that are predominantly expressed from the paternal *P. ficus* genome (PB, 0.05 < *p_m_* < 0.35) and two completely paternally expressed genes (P, *p_m_* ≤ 0.05).

In the reproductive tract, ASE patterns are significantly different to those found in soma (*G*-test of independence, *G* = 3,700.5, df = 4, *P* < 0.001) and even more shifted toward the maternal genome, with >99% genes showing a predominance of maternal expression: 80.5% (4,472) are completely M, and 18.8% (1,044) MB. Only 0.5% of genes expressed in the reproductive tract (30) were classified as B, while we found 8 and 2 genes to be, respectively, PB and P.

The observed differences between tissues are indicative of different patterns of ASE in soma and reproductive tract. We also analyzed differences in ASE for the 4,005 overlapping genes which were expressed in both soma and reproductive tract and found only moderate correlation (Spearman’s *ρ* = 0.50) between *p_m_* estimates in both tissues (fig. 1*D*). Only 34.5% belong to the same ASE category in both tissues (fig. 1*E*), which is indicative of changes in imprinting status between somatic and reproductive tissues, with paternal chromosomes contributing more to transcription in the soma.

### Parent-of-Origin Expression in Intraspecific Males

**Fig. 2.**
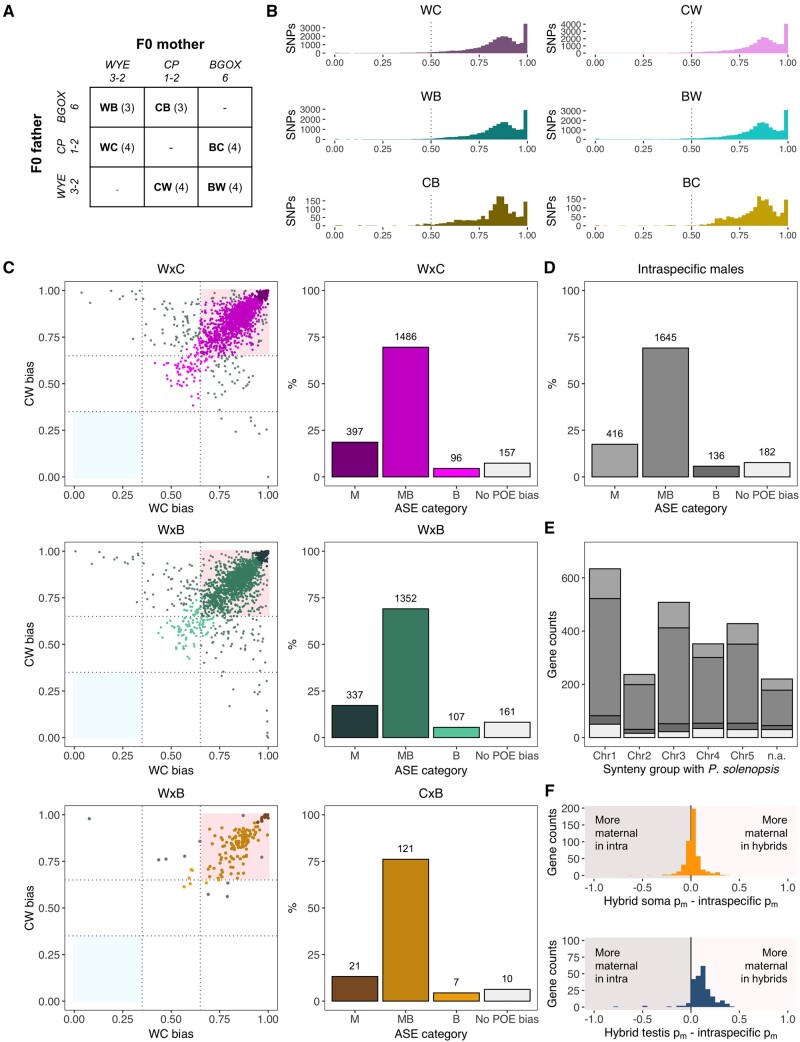
Quantification of parent-of-origin ASE in F1 pure *P. citri* males from pairs of reciprocal crosses between three isofemale lines. (*A*) Cross scheme (number of replicates in brackets) (*B*) Histogram of expression biases to maternal genome, *p_m_*, at SNP level in all six genotypes (pooled maternal and paternal counts between replicates). The dotted line indicates complete biparental expression. (*C*) Gene *p_m_* scatterplots in both reciprocal genotypes from each parental cross pairs (left panels). Maternally biased genes are expected in the top right corner (pink), while paternally biased genes are expected in the bottom left panel (light blue). Genes with discordant *p_m_* in reciprocal genotypes (not showing a true parent-of-origin ASE pattern) are shown in gray. Gene counts by ASE categories are shown in the right panels. (*D*) Final counts of genes with ASE information in at least one pair of genotypes after combining data from all parental cross pairs. (*E*) Distribution of ASE expression categories across the genes that could be mapped to *Phenacoccus solenopsis* chromosomes. (*F*) Histogram of *p_m_* differences between soma-only genes (top panel) and reproductive tract-only genes (bottom panel) in CF hybrids and intraspecific males (*p_m_* averaged across all three parental cross pairs).

In order to confirm that the global bias to the maternal genome observed in CF males is due to parent-of-origin effects (which cannot be determined without the reciprocal cross), and to rule out that this extreme expression pattern is a product of hybridization, we also estimated ASE in pure *P. citri* males produced in reciprocal crosses between three isofemale lines (fig. 2*A*).

We found 24,660 high-confidence SNPs shared with a read depth of at least 20× in the transcriptomes of 4 WC and 4 CW replicates originated in reciprocal crosses between WYE3-2 and CP1-2 (WxC), 20,903 SNPs in 3 WB and 4 BW replicates from WYE3-2 × BGOX-6 (WxB) crosses and only 1,592 SNPs in 3 CB and 4 BC replicates from CP1-2 × BGOX-6 (CxB) crosses (supplementary fig. S3, Supplementary Material online) (107–218 M reads per sample). As in hybrid crosses, *p_m_* distribution across SNPs shows a strong bias toward the maternal genome in all genotypes (fig. 2*B*), with averages ranging between 0.82 and 0.85 (0.14–0.15) (supplementary table S2, Supplementary Material online). We assigned 16,748 exonic SNPs (68% of total) to 2,136 genes in WxC replicates, 14,775 (71%) to 1,957 genes in WxB replicates and 1,054 (66%) to 159 genes in CxB replicates (6.8 SNPs per gene in WxC and WxB crosses and 5.7 in CxB crosses). Assignment of genes to ASE categories for each of the six intraspecific genotypes individually revealed similar patterns to those observed in CF soma, with a predominance of MB and M genes (supplementary fig. S4, Supplementary Material online).

Actual parent-of-origin ASE patterns could be estimated by crossing *p_m_* estimates between each pair of reciprocal genotypes (fig. 2*C*). Average *p_m_* differences between reciprocal crosses are small in all three pairs (|0.055|–|0.065|), with 85–87% of genes belonging to the same ASE category in both cross directions. Incorporating reciprocal information allowed us to determine that only 6–8% of genes show allelic biases that are not consistent in both cross directions and therefore do not represent true parent-of-origin effects (“no POE bias”). These genes include all those that had been classified as predominantly paternally expressed (PB or P) in individual intraspecific genotypes, suggesting that the putatively PB and P genes found in hybrid soma and reproductive tract do not represent true parent-of-origin expression and are most likely line-specific effects.

Among the genes with consistent parent-of-origin effects in intraspecific crosses, 69–76% exhibit MB expression and a further 13–19% are completely M, with only 4–5% showing biparental expression. These patterns are consistent across all three intraspecific crosses (*G*-test of independence, *G* = 7.702, df = 6, *P* = 0.26), which allowed us to combine data from all three pairs of genotypes and assign 2,379 genes with ASE information in at least one genotype to a parent-of-origin expression category at the intraspecific level (fig. 2*D*). To investigate patterns of genetic linkage in relation to parent-of-origin expression, we took advantage of synteny alignments of the PCITRI.V0 assembly to a chromosome-level assembly of another closely related mealybug, *Phenacoccus solenopsis* (Li et al. 2020)*.* Genes belonging to each ASE category (including genes with no true POE bias) are homogeneously distributed across all five synteny groups to *P. solenopsis* (*G*-test of independence, *G* = 13.485, df = 12, *P* = 0.33), revealing a lack of chromosomal biases in parent-of-origin expression (fig. 2*E*).

Finally, we examined the correspondence between the estimated ASE patterns in CF hybrids and intraspecific males (fig. 2*F*). *p_m_* estimations show high consistency (Spearman’s *ρ* = 0.83) for 461 soma-limited genes (i.e., present in intraspecific and CF soma data sets but not in CF reproductive tract), but only moderate for 231 reproductive tract-limited genes (*ρ* = 0.35), which showed a higher bias to the maternal genome in hybrids.

Overall, the data in both hybrids and intraspecific crosses reveal a consistent genome-wide parent-of-origin, tissue-specific global expression bias to the maternal genome consistent with cytogenetic observations of paternal genome heterochromatinization (see below), albeit with partial transcriptomic activity of paternal chromosomes.

### Cytological Evidence of Paternal Genome Silencing in the Male Reproductive Tract

**Fig. 3.**
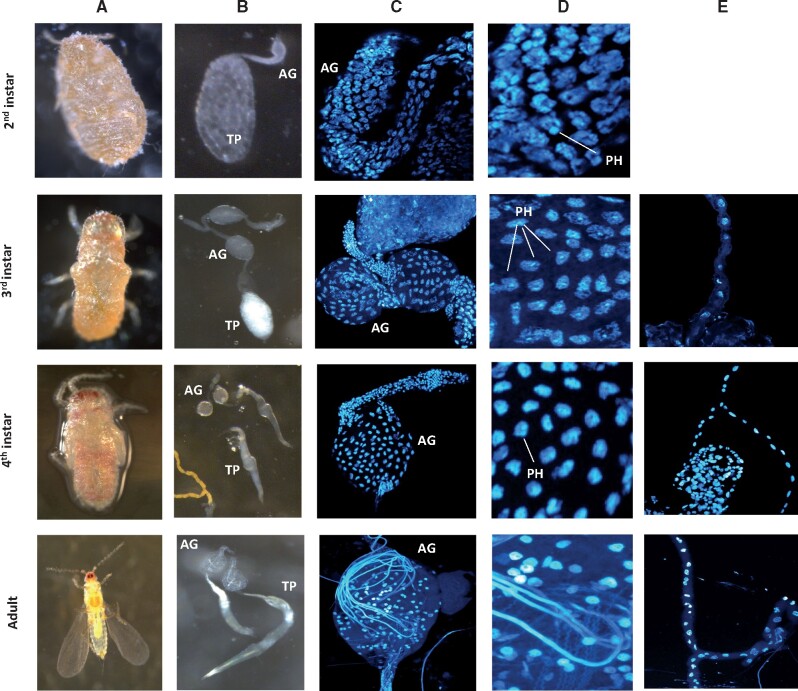
Cytogenetic analysis of paternal genome heterochromatization in cells of the accessory glands and testis proper and Malpighian tubes at four stages of development: second instar, third instar, fourth instar and adult. AG, accessory gland; TP, testes proper; PH, paternal genome heterochromatization. (*A*) Images taken on a dissection microscope of the individual from which the reproductive tract and Malpighian tubes were dissected. (*B*) Images taken on a dissection microscope of the dissected testes. (*C*) Confocal image of the accessory gland stained with DAPI (×63). (*D*) Enlarged section from image (*C*) showing PH status in the AG. (*E*) Confocal image of the Malpighian tubes stained with DAPI (×63).

We complemented our transcriptomic analysis of allele-specific gene expression with a cytological time series of paternal genome condensation dynamics in *P. citri* males in successive larval stages (fig. 3), with a focus on the reproductive tract. In mealybugs, spermatogenesis begins at second larval instar (Nur 1962; Bongiorni et al. 2004; Bain 2019). The reproductive tract consists of a pair of elongated testes—“testes proper”—located at the base of two accessory glands which lead up to the ejaculatory duct (Nur 1962). At early second larval instar, the dense nuclear bodies which result from heterochromatinization of paternal chromosomes are widespread in the testes (supplementary fig. S5*A*, Supplementary Material online). Condensed paternal chromosomes can also be first observed in the rapidly dividing somatic cells of the developing accessory glands in the second larval instar (fig. 3*B*, *D*), coinciding with early spermatogenesis in the testis. During third instar, coinciding with late spermatogenesis, all cells of the fully developed accessory glands clearly show heterochromatinization of paternal chromosomes. Interestingly, however, during fourth instar paternal chromosome heterochomatinization can only be observed in a fraction of the cells of the accessory glands, and the signal is completely lost in adults. In contrast, detecting the heterochromatic state of paternal genomes in the testis proper after second instar is harder, since there are very few cells besides spermatocytes. The exception are cyst wall cells lining sperm cysts, which only occasionally show heterochromatic bodies in third instars (supplementary fig. S5*B*, *C*, Supplementary Material online). In addition to the reproductive tract, we also targeted Malpighian tubules, which have been shown to lack paternal genome heterochromatinization in larval male instars (Nur 1967). The dynamic patterns of paternal chromosome silencing and decondensing in the male reproductive tract are in stark contrast to Malpighian tubules, which consistently lack or show very limited paternal chromosome heterochromatinization across all male stages (fig. 3*E*).

Since our ASE patterns are consistent with strong paternal chromosome heterochromatization, we speculate that there is very limited transcription taking place in the adult testis (as spermatogenesis has already finished in adults) and our RNA-sequencing captured residual gene expression from earlier stages in the reproductive tract.

### Functional Investigation of Genes without Maternal Expression

We then examined the functional profile of the minority of genes exhibiting biparental or predominantly paternal expression in hybrids and intraspecific males identified in our ASE analysis. In hybrid soma, where our detection power is highest thanks to more genes with allele-specific information, 172 B, PB, and P genes have at least a GO term assigned, against a background population of 3,193 genes with ASE information and GO assignment. We found four GO terms significantly enriched in biparental and paternally biased genes (FDR < 0.05): translation, oxidation–reduction process and ribosome/structural component of ribosome (supplementary table S3, Supplementary Material online). We did not find significant enrichment for any GO terms in hybrid testis (172 B, PB, and P genes GO-annotated vs. a population of 1,535) or intraspecific males (77/1,231).

We further examined putative functions of biparentally expressed genes using BLAST homology searches. We found genes with mitochondrial functions to be common among B and PB genes in hybrid soma (100/292 annotated genes, supplementary data set S1, Supplementary Material online) and intraspecific (29/108, supplementary data set S2, Supplementary Material online). These include genes that code for structural components of mitochondrial ribosomes (for example, in hybrid soma, six small subunit proteins, *mRpS2*, mRpS12, mRpS15,*mRpS23*,*mRpS29* and *mRpS30*, and 19 large subunit proteins: mRpL1,*mRpL3*,* mRpL4*,* mRpL9*,* mRpL10*,* mRpL12*,* mRpL18*,* mRpL19*,* mRpL20*,* mRpL22*,* mRpL24*,* mRpL27*,* mRpL28*,* mRpL32*,* mRpL37*,* mRpL38*,* mRpL39*,* mRpL43,* and *mRpL52*) and members of the mitochondrial respiratory chain, such as homologs to *Drosophila melanogaster ND-18*,* ND-20, ND-23*,* ND-30*, *ND-39*,* ND-49*, *ND-51*,* ND-B14*, and *ND-PDSW* (NADH: ubiquinone oxidoreductase complex I), ATPsynF and ATPsynO (mitochondrial ATP synthase complex V) or Cyt-c-p (cytochrome c). We also found several B genes related to glucose metabolism (6-phosphofructokinase, inositol-3-phosphate synthase, ribulose-phosphate-3-epimerase) and fatty acid metabolism (fatty acid synthases, short-chain synthetase and ligase, long-chain ligase and dehydrogenase) in both hybrid soma and intraspecific males. In contrast, these genes with mitochondrial functions identified in hybrid soma and intraspecific males were found to be M or MB in the hybrid reproductive tract, where only 25 B genes could be annotated (supplementary data set S3, Supplementary Material online). Among these genes, we did not identify any candidates involved in reproductive functions.

Since many of the above B genes identified hybrid soma and intraspecific males are constitutive, we speculated that biparentally expressed genes would tend to exhibit higher expression levels than completely maternal genes. To assess the effect of bias to the maternal genome in gene expression levels, we fitted a linear model with log-transformed TPM counts as the response variable and *p_m_* and the quadratic term *p_m_*^2 as fixed effects. In order to obtain independent estimates of gene expression levels and bias to the maternal genome, we used gene TPM counts estimated from three additional whole adult *P. citri* transcriptomes (see Materials and Methods) and *p_m_* estimates obtained from intraspecific F1 males. We found both a significant linear and quadratic relationship between *p_m_* and gene expression, albeit with a very low effect size (*R*^2^ adjusted = 0.026, *F*_2,2376_ = 33.42, *P* < 0.001, supplementary fig. S6, Supplementary Material online).

**Fig. 4.**
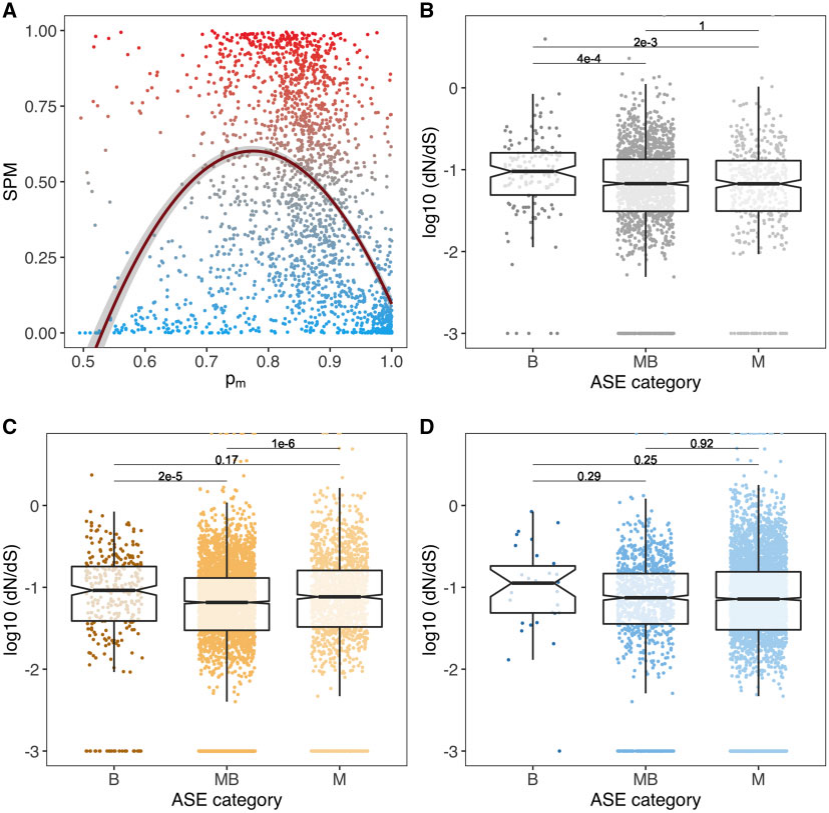
Sex-specific expression and evolutionary rates of genes with consistent parent-of-origin information in intraspecific males. (*A*) Scatterplot of sex-specificity and bias to the maternal genome *p_m_* for all 2,197 genes with consistent POE patterns. Transcriptomes of *Planococcus citri* males and females from the WYE3-2 line were used to estimate sex-specificity (SPM) (blue dots represent male-biased genes; gray, nonbiased genes; red, female-biased genes). The regression curve predicted by a GLM exploring the relationship between sex-specificity and bias to the maternal genome (SPM ∼ *p_m_* + *p_m_*^2) is shown in dark red. (*B*) Boxplot showing differences in evolutionary rates (as log-transformed d*N*/d*S* ratios) between biparental, maternally biased and maternal-only expression in intraspecific crosses. The *P*-values of post hoc Nemenyi tests in pairwise comparisons between ASE categories are shown. To plot genes with d*N*/d*S* = 0, *a* 0.001 constant was added to all d*N*/d*S* values (*C*) d*N*/d*S* ratios between biparental, maternally biased and maternal-only genes in hybrid soma. (*D*) d*N*/d*S* ratios between biparental, maternally biased and maternal-only genes in the hybrid reproductive tract.

Also, in addition to measuring parent-of-origin expression, our RNA-sequencing design allowed us to examine more straightforward sex-specificity of gene expression. We evaluated whether biparental genes are more male-specific than genes with higher degree of maternal expression using a specificity metric (SPM)—a proportion of expression ranging from 0 (male-specific) to 1 (female-specific)—calculated from transcriptomes of whole *P. citri* males and females (Materials and Methods). We found an extreme distribution of sex-biased genes in the *P. citri* transcriptome (supplementary fig. S7, Supplementary Material online), which might be driven by the extreme sexual dimorphism in mealybugs. Then, we fitted a quasibinomial GLM to explore the relationship between SPM and parent-of-origin expression in intraspecific F1 males. Both *p_m_* (*F* = 5.25, df = 1, *P* = 0.022) and *p_m_*^2 (*F* = 734.72, df = 1, *P* < 0.001) were significant, indicating that both biparental and completely maternal genes tend to be more male-specific than genes showing an intermediate degree of bias to the maternal genome (fig. 4*A*). To rule out that the trend toward lower male-specificity at intermediate degrees of maternal genome bias could be driven by background expression of female-specific genes in males, we refitted the model twice after removing genes with low (TPM < 10) and moderate expression levels (TPM < 100) (supplementary fig. S8, Supplementary Material online). The results of these new models were consistent with the original GLM (without TPM < 10: *p_m_*, *F* = 5.86, *P* = 0.016 and *p_m_*^2, *F* = 659.20, *P* < 0.001; without TPM < 100: *p_m_*, *F* = 10.81, *P* = 0.001 and *p_m_*^2, *F* = 277.82, df = 1, *P* < 0.001).

### Rates of Molecular Evolution between Biparental and Maternal Genes

Our interspecific crossing design presented an opportunity to examine long-term evolutionary rates of genes with different parent-of-origin expression patterns. We aligned *P. ficus* sequences to the *P. citri* reference genome to obtain variants and annotated the effects (i.e., synonymous or nonsynonymous) with a pipeline described in Materials and Methods. Due to the inconsistencies in ASE bias between reciprocal crosses for some genes (“no POE bias”), we only considered biparental genes with consistent parent-of-origin effects in intraspecific F1 males. In these, we found a significant effect of expression type (B, MB, or M) on rates of molecular evolution (d*N*/d*S*) using a nonparametric Kruskal–Wallis test (*χ*^2^_2_=14.74, *P* = 0.0006). Using a post hoc Nemenyi test, this difference is driven by elevated d*N*/d*S* in biparentally expressed genes, which evolve faster than maternally biased (*P* = 0.0004) or maternal-only (*P* = 0.002) genes. The two classes of maternally skewed genes do not evolve differently from each other (*P* = 1.000) (fig. 4*B*).

We also repeated these analyses with parent-of-origin expression in soma and reproductive tract in CF males, to examine if these differences in evolution rates were also found in hybrids and specifically in reproductive tissues. While we also found a significant effect of expression class in d*N*/d*S* ratios in hybrid soma (*χ*^2^_2_ = 40.23, *P* < 0.001), biparental and maternal-only genes both evolve faster than maternally biased genes but not differently from each other (fig. 4*C*). In the hybrid reproductive tract, however, biparental, maternally biased and maternal only genes do not show significant differences in their evolution rates (*χ*^2^_2_ = 2.62, *P* = 0.270) (fig. 4*D*).

## Discussion

Mealybugs have a unique reproductive strategy, PGE, where males inherit a haploid genome from both parents, but the paternally inherited chromosomes are eliminated during spermatogenesis and excluded from viable sperm. This type of reproduction is thought to both originate from and lead to strong intragenomic conflict between maternally and paternally inherited alleles. The recurring evolution of transcriptional repression of the paternal genome in males across three of the seven independent origin of PGE (Burt and Trivers 2006; Gardner and Ross 2014; Hodson et al. 2017) has been interpreted as a maternal genome strategy to ensure the transmission advantage through males gained from PGE, preventing the paternal genome from expressing adaptations to fight its exclusion from sperm in an evolutionary arms race over transmission (Herrick and Seger 1999; Ross et al. 2010). However, our understanding of PGE silencing is still limited and had not yet been studied on a genome-wide scale in a species with this reproductive system.

We show that gene expression does indeed show a global expression bias toward maternally inherited alleles in both soma and reproductive tract: we estimated ASE profiles for >5,500 genes in hybrid mealybug males originated from crosses between *P. citri* females and *P. ficus* males, and found evidence of bias toward the maternal genome in up to ∼95% genes in soma and ∼99% in reproductive tract. This data alone cannot prove parent-of-origin imprinting, since we could not include males from the reciprocal cross to examine lineage-of-origin effects or other artifacts (Wang and Clark 2014). It is unclear why hybrid males from the reciprocal cross suffer more strongly from hybrid incompatibilities. Mealybugs do not have differentiated sex chromosomes (Hughes-Schrader 1948; Li et al. 2020), but since males are to a great extent hemizygous, Haldane’s rule might explain the differential mortality between hybrid sexes (Koevoets and Beukeboom 2009). In any case, we complemented these results with a subsequent analysis of male offspring from reciprocal intraspecific *P. citri* crosses, where >2,100 genes reflected the strong bias to the maternal genome observed in viable hybrids. We find that ASE profiles are extremely consistent not only between biological replicates but also across hybrids and intraspecific genotypes.

Thus, we conclusively show a strong parent-of-origin effect in gene expression (genomic imprinting) in mealybugs, whereby maternal chromosomes are the main contributors to transcriptomic activity in males. Although we did not target females in this study, the wealth of evidence showing that females in mealybugs and other germline PGE species express genes in a Mendelian fashion and always lack both the conspicuous heterochromatization and enrichment of heterochromatin-associated histone modifications observed in males (Schrader 1921; Brown and Nelson-Rees 1961; Brown and Nur 1964; Brown and Wiegmann 1969; Brown 1972; Brun et al. 1995; Borsa and Kjellberg 1996; Bongiorni et al. 2001; Hodson et al. 2017; Li et al. 2020) indicates that the genome-wide imprinting under PGE is male-specific.

However, we also find that the paternal genome is not completely inert in males: paternally inherited alleles are expressed to some extent for ∼75–80% of genes in hybrid soma and whole intraspecific males. This proportion is lower in the reproductive tract, where only ∼20% of genes are expressed with some contribution of paternal copies. These differences between the soma and the reproductive tract suggest that paternal alleles are expressed in a tissue-specific manner: for example, many genes with exclusive maternal allelic expression in the reproductive tract show incomplete paternal genome silencing in the soma.

The partial transcriptional activity of the paternal genome might be due to tissue or cell-specific loss of heterochromatinization of paternal chromosomes. In mealybug males, the paternally inherited chromosome set becomes highly condensed during midcleavage stage in all embryonic nuclei (Bongiorni et al. 2001). The condensed paternal set is enriched with heterochromatin-associated proteins and histone modifications, such as the H3K9me3-HP1 (heterochromatin protein 1) and H3K27me3-PRC2 (polycomb repressive complex 2) pathways (Bongiorni et al. 2001, 2007; Bain 2019). Interestingly, the maintenance of the heterochromatic state of the paternal genome depends directly on the presence of the maternally inherited set (Nur 1962; Chandra 1963; Brown and Nur 1964). However, in a small number of male tissue types (such as the Malpighian tubules), paternal genome heterochromatinization is lost in later life stages (Nur 1966, 1967, 1970). This is compatible with the expression patterns that we observe in our analysis: for example, genes showing an intermediate degree of bias toward the maternal genome might be expressed in cells both with and without paternal genome silencing, while biparentally expressed genes are probably specific to tissues without heterochromatinization or sufficiently enriched in those tissues to dominate the expression signal.

Alternatively, it is also possible that some genes are expressed from heterochromatic chromosomes. This is the case in the heavily condensed Dot chromosome in *Drosophila* (Riddle and Elgin 2018) and under X-inactivation in mammals—also achieved via heterochromatinization (Lyon 1961; Wutz 2011)—where a small proportion of genes (1–3% in mouse, 15% in human) on the X escape inactivation (Carrel and Willard 2005; Filippova et al. 2005; Heard and Disteche 2006; Yang et al. 2010; Crowley et al. 2015). This might also occur in mealybugs if the regulation of histone modifications is leaky for genes with partial contribution of paternal chromosome transcription, albeit probably not at the large scale found in this study since there is direct evidence of RNA synthesis inhibition from the heterochromatic set (Berlowitz 1965). Further mechanistic insight on gene regulation within heterochromatic chromosomes and establishment and maintenance of chromatin state will be key to determine how paternal chromosomes contribute to gene expression.

The more extreme parent-of-origin expression pattern in the reproductive tract, the arena where elimination of paternal chromosomes takes place, is particularly interesting to consider in light of intragenomic conflict and the arms race hypothesis between parental genomes. Under such an arms race, two opposite patterns can be predicted (Herrick and Seger 1999; Ross et al. 2010): the reproductive tract could either be a hotspot for paternal chromosome reactivation—as a result of successful anti-PGE adaptations—or a tissue in which the control exerted by the maternal genome over the paternal is most stringent—to prevent such responses. Our analysis showing stronger paternal genome silencing in the reproductive tract is consistent with the latter coevolutionary stage. However, it must be noted that male meiosis and the subsequent degradation of spermatids containing paternal chromosomes mostly take place prior to adulthood, during second/third larval instars (Nur 1962; Bongiorni et al. 2004; Bain 2019). By targeting adult males for RNA-seq, we might have missed paternally expressed genes acting during or just prior to meiosis that could indicate a different scenario. We show cytological evidence of widespread heterochromatinization of paternally inherited chromosomes in early second instar testes and the somatic part of the reproductive tract of third instar males—but, unfortunately, how ubiquitous it is in the germline part of the testis is less clear, due to the fact that germline cells undergo rapid cell division. Therefore, future RNA-seq analyses of these developmental stages would allow capturing spermatogenesis and framing paternal genome expression patterns directly in the temporal context of their elimination.

Of course, there might be another simpler, mechanistic explanation for the stronger paternal genome silencing in the reproductive tract that does not invoke intragenomic conflict: that expression of maternal alleles would be inevitably predominant in the reproductive tissues due to the physical elimination of paternal chromosomes in spermatogenesis. However, since the paternal genome is only eliminated in secondary spermatocytes during meiosis II, this would necessarily imply that the signal is dominated by transcriptional activity of the packaged haploid set in spermatids (which is unlikely; see e.g., Ren et al. 2017), rather than by transcriptional dynamics of active nuclei where the paternal chromosomes are still present.

Another alternative hypothesis is that a less strict paternal genome silencing in somatic tissues might have been selected for to increase transcription of particular genes by recruiting the contribution of paternal gene copies (Nur 1966). We find that biparentally expressed genes tend to be more highly expressed than maternally imprinted genes in *P. citri* males. Also, in hybrid soma (where our power to detect ASE is highest), biparental genes are enriched in fundamental processes, such as translation and oxidation–reduction. Many of these genes play important roles in the mitochondria: for example, nuclear-encoded mitochondrial ribosome proteins (MRPs; Richman et al. 2014; Tselykh et al. 2005; Rackham and Filipovska 2014) and others involved in the electron transfer chain, suggesting that reaching diploid expression levels could be important if mitochondrial function is limiting. Of course, a possible reason for the overrepresentation of MRPs genes might be their accelerated evolution rates (O’Brien 2003; Desmond et al. 2011), which facilitates estimation of ASE thanks to higher SNP density. Other biparental genes are involved in glucose and fatty acid metabolism. Mealybug males cease to feed at late second instar (Franco et al. 2009), so upregulating transcription of gene pathways dedicated to energy production could be a mechanism to compensate for dietary restriction. Tellingly, one of the cell types where paternal genome heterochromatinization is lost in *P. citri* are oenocytes (Nur 1966), which specialize in lipid storage and metabolism (Makki et al. 2014).

We note that this putative dosage compensation mechanism is not incompatible with the intragenomic conflict hypothesis: the maternal genome may be firmly in control of paternal genome reactivation, allowing its expression only for subsets of genes that are important for male function and do not pose a risk to the interests of the maternal genome (i.e., loci at which conflict is less likely to arise). This could explain why biparental genes are much more scarce in the reproductive tract, where scope for conflict is highest. Alternatively, there simply might be less stringent selection on silencing in somatic tissues, and therefore biparental expression might be driven by other selective forces or simply random, transitory errors in gene silencing. However, we found that biparentally expressed genes are faster evolving (i.e., have higher d*N*/d*S* ratios) than genes with predominantly maternal expression in intraspecific males; such a pattern implies consistently different expression patterns over evolutionary time and is therefore difficult to reconcile with stochastic failures of the gene silencing machinery. Interestingly, this is not the case in the reproductive tract of hybrid males, where genes apparently evolve at the same rate—although our ability to detect differences in d*N*/d*S* rates might be hindered by the limited number of biparentally expressed genes identified there (which is in itself suggestive of differences in strength of intragenomic conflict between soma and reproductive tract).

The implications of differences in evolutionary rate are difficult to parse with the current data. For instance, if paternal genes escaping silencing are indeed in an evolutionary arms race with the maternal silencing machinery, the faster evolution could be driven by positive selection of novel variants. However, faster evolutionary rates do not per se imply adaptation, as such patterns can easily arise from the opposite: relaxed selection, as has been argued for genes involved in reproduction (Dapper and Wade 2016). As such, evidence for ongoing conflict is weak from these data alone, but at the same time cannot be ruled out. As an alternative, it is worth noting that genes with maternally biased and, especially, strictly maternal expression are necessarily expressed in the haploid state. As such, any deleterious variation, even that which would otherwise be recessive, is exposed to selection in males, resulting in strong purifying selection (Gerstein et al. 2011). From this perspective, the apparent elevation of d*N*/d*S* of biparentally expressed genes in intraspecific males might be more correctly viewed as a decrease in d*N*/d*S* of maternally biased genes, due to an increased role of purifying selection. In either case, it will require more careful scrutiny of within-species variation to obtain estimates of adaptive evolution across parent-of-origin-bias categories and disentangle these alternative explanations.

## Conclusions

In this study, we show genome-wide parent-of-origin expression in an insect whereby maternally inherited chromosomes are the main contributors to transcriptomic activity in males. In recent years, a number of studies have considered genomic imprinting beyond mammals and flowering plants (de la Casa-Esperón 2012; MacDonald 2012). We now have several allele-specific studies of imprinted gene expression (or lack thereof) in insects (Coolon et al. 2012; Kocher et al. 2015; Wang et al. 2016; Galbraith et al. 2016; Marshall et al. 2020). Yet most of these show very limited evidence for genomic imprinting, so our understanding of this process in insects remains scarce. So far, most studies have focused on eusocial Hymenoptera, specifically to test the kinship theory of genomic imprinting, (Queller 2003; Patten et al. 2014). However, the interactions between relatives with asymmetric genetic relationships that underlie this theory are not exclusive to classic haplodiploidy and eusociality. PGE taxa share the same genetic relationships between relatives as those found in true haplodiploids, but unlike in classic haplodiploidy males are still diploid and carry a complete haploid copy of their father’s genome, which is not transmitted to the offspring. As a result, there might be conflict between maternally and paternally inherited alleles over transmission—and over male fitness in scenarios of sibling competition, as paternally inherited alleles in males might favor the fitness of female relatives over their own (Ross et al. 2011).

From an intragenomic conflict perspective, the differential parent-of-origin-specific expression patterns between somatic and reproductive tissues of mealybug males found in this study are consistent with a coevolutionary scenario over maintenance of paternal silencing in which the maternal genome appears to have stringent control over the paternal set, especially in the reproductive tract. However, there are other potential explanations for these patterns that cannot be excluded. Of course, our understanding of the interactions between parental genomes will be incomplete without also characterizing the molecular mechanisms that allow the dynamic spatiotemporal regulation of paternally inherited chromosomes and, ultimately, their germline elimination. The prime candidate in mealybugs is DNA methylation, which differs remarkably between both sexes in *P. citri* (Bain et al. 2020), although current evidence of differential allele-specific methylation in males is contradictory (Bongiorni et al. 1999; Buglia et al. 1999). However, it is unclear how this signal interacts with heterochromatin-associated epigenetic modifications, and whether other players, such as noncoding RNAs, might also play a role. Finally, another exciting prospect for this and future analysis strategies is their application to other germline PGE taxa with and without paternal chromosome silencing, in order to gain a comparative understanding of the somatic manifestations and epigenetic regulation of this bizarre genetic system and its evolutionary implications.

## Materials and Methods

### Experimental Crosses

Hybrid crosses were conducted between individuals from wild-derived, highly inbred laboratory strains: WYE3-2 (*P. citri*, derived from an English population, >25 generations of sib-mating) and PF1-1 (*P. ficus*, derived from an Israeli population, >10 generations of sib-mating). Due to extreme male specific mortality in hybrid offspring of crosses between *P. ficus* females and *P. citri* males, we were unable to raise viable adult male hybrids from these crosses. Therefore, only hybrid males from *P. citri* mothers and *P. ficus* fathers (CF crosses) could be sequenced. Intraspecific crosses were produced in all possible combinations between WYE3-2 and two additional isofemale *P. citri* lines, CP1-2 (derived from Israel) and BGOX-6 (derived from England) (>40 generations of sib-mating). All six reciprocal genotypes (WC, CW, WB, BW, CB, BC) yielded viable adult males.

Mealybugs were reared on sprouted potatoes placed on tissue paper in sealed plastic stock bottles and kept at 25 °C and a 16 h-light/8 h-dark photoperiod without humidity control. Males and females used in experimental crosses were isolated before sexual maturity and isolated until adulthood. Hybrid crosses were set by placing pools (10–20 individuals) of brothers from the paternal species and sisters from the maternal species in 6 cm-diameter glass Petri dishes. To encourage mating, a filter paper impregnated with 10 ng of synthetic sex pheromone from the paternal species (Bierl-Leonhardt et al. 1981; Hinkens et al. 2001) was placed in the Petri dish. After all males in the Petri dishes died, females were transferred to rearing bottles in groups to lay eggs. Hybrid F1 offspring were reared until becoming sexually differentiated (third instar). At that stage, viable CF males were transferred to glass vials sealed with cotton wool to reach adulthood. For intraspecific crosses, individual mating pairs were set in glass vials containing a single potato sprout. Successful matings were evidenced by females laying eggs 2–5 days postmating, which were transferred to individual bottles. Males were reared and isolated in glass vials until adulthood.

### DNA and RNA Extraction and Sequencing

To sequence the genomes of the parental lines used in CF crosses, genomic DNA was extracted from 5 to 10 adult WYE 3-2 (*P. citri*) and PF1-1 (*P. ficus*) virgin females. Sample lysis, proteinase K digestion and RNA removal were performed using a DNeasy Blood & Tissue kit (Qiagen, The Netherlands) and isolation of gDNA was carried out with a Wizard Genomic DNA Purification Kit (Promega) according to manufacturer’s instructions. A single TruSeq library (350 bp insert size) for *P. citri* and two TruSeq libraries (350 bp and 550 bp insert sizes) for *P. ficus* were generated and sequenced on a HiSeq 2500 instrument by Edinburgh Genomics (The University of Edinburgh, UK). For intraspecific crosses, genomic DNA was extracted using a custom protocol from 6 to 8 virgin females from WYE3-2, CP1-2, and BGOX-6 sisters to the females used in the crosses and TruSeq DNA PCR free gel library (350 bp insert size) was sequenced on a HiSeq X instrument (150-bp read pairs).

For RNA-seq of CF males, we extracted RNA from somatic and reproductive tissues of >70 adult F1 male offspring from three pools of *P. citri* sisters mated to *P. ficus* males. Males were dissected in RNAlater (Thermo Fisher Scientific), and soma and reproductive tract were immediately transferred to ice-cold TRIzol (Invitrogen) and stored at –80 °C. RNA was extracted using isopropanol and chloroform (2.5:1) and linear acrylamide as a carrier. After extraction, residual gDNA digestion was performed using DNAse I (Thermo Fisher Scientific) and RNA samples were purified with RNA Clean & Concentrator-5 (Zymo Research). Due to low RNA yields, cDNA amplification was performed using the Ovation RNA-Seq System V2 (NuGen). Two independent cDNA amplifications from each sample were performed separately to be sequenced as technical replicates. cDNA samples were purified using MinElute Reaction Cleanup Kit (QIAGEN, The Netherlands) in TE buffer. In total, 12 Illumina TruSeq Nano libraries (350 bp insert size) were generated and sequenced on two lanes on a Illumina HiSeq 4000 instrument (75 bp paired-end reads) by Edinburgh Genomics.

We also generated three Illumina TruSeq stranded mRNA-seq libraries from three pools of whole adult *P. citri* males and females from the maternal line used in the hybrid crosses (WYE3-2). The first male and female transcriptomes were sequenced on the Illumina HiSeq 4000 instrument (75 bp paired-end reads) and the remaining two on the Illumina NovaSeq S2 instrument (50 bp paired-end reads).

For intraspecific crosses, RNA was extracted from pools of 20–50 flash frozen full F1 brothers descending from a single cross using the PureLink RNA purification kit with DNase I digestion (Thermo Fisher Scientific). TruSeq stranded mRNA-seq libraries (350 bp insert size) were generated from 23 samples (4 WC, 4 CW, 3 WB, 4 BW, 4 CB, and 4 BC) and sequenced on a single lane of NovaSeq S2 instrument (50 bp paired-end reads) by Edinburgh Genomics.

DNA and RNA samples were quantified using Qubit BR Assay Kit (Thermo Fisher Scientific) and their integrity was assessed via 1% agarose gel electrophoresis or Bioanalyzer RNA 6000 Nano kit (Agilent). Library quantification, normalization and quality control were performed by Edinburgh Genomics (The University of Edinburgh, UK).

### SNP Calling

To call discriminant SNPs between *P. citri* and *P. ficus*, we mapped 35.8 M *P. citri* read pairs (16× coverage) and 123.3 M *P. ficus* read pairs (40× coverage) to our reference *P. citri* genome (PCITRI.V0) with bwa 0.7.15-r1140 (BWA-MEM algorithm) (Li 2013). We called a raw set of variants using FreeBayes v1.2.0 (Garrison and Marth 2012) with the following settings: --haplotype-length 0 --standard-filters --min-alternate-fraction 0.05 -p 2 --pooled-discrete --pooled-continuous. We used vcffilter (https://github.com/vcflib/vcflib#vcffilter, last accessed March 02, 2021) to filter the resulting VCF file (“DP > 10 & SAF > 2 & SAR > 2 & RPR > 1 & RPL > 1”) and discard all non-SNP variants. We obtained an initial set of 5,288,538 discriminant SNPs between both genomes using the SelectVariants walker in GATK v3.8 (McKenna et al. 2010) to keep only variants that were monomorphic for an alternative allele in *P. ficus* and for the reference allele in *P. citri.* To keep only high-confidence sites, we then filtered out sites with AO < 20 and AO/DP < 0.99 to yield a final set of 4,670,197 between-species discriminant SNPs.

The same strategy was followed to call discriminant SNPs between the three pairs of isofemale *P. citri* lines used in intraspecific crosses. We mapped 183.7 M read pairs for WYE3-2, 163.8 M for CP1-2 and 325.0 M for BGOX-6 to the reference PCITRI.V0 (81×, 77×, and 135× coverages, respectively), called variants with freebayes and filtered the resulting VCF file as above. Then, we generated six separate VCF files (two for each pair of lines) in which the first line was monomorphic for the reference allele and the second was monomorphic for an alternate allele with DP > 10 in both. In total, we obtained 806,554 discriminant SNPs between WYE3-2 and CP1-2, 837,922 between WYE3-2 and BGOX-6 and only 57,154 between CP1-2 and BGOX-6.

### RNA-Seq Mapping and ASE Estimation at Discriminant SNPs

To estimate ASE patterns in male transcriptomes, we mapped RNA-seq reads to custom pseudogenomes for each hybrid and intraspecific cross generated by hard-masking SNP positions with different parental alleles.

Initial quality control and read trimming of raw sequencing data were performed with FastQC v0.11.5 (https://qubeshub.org/resources/fastqc, last accessed March 02, 2021) and Trimmomatic v0.36 (Bolger et al. 2014) with default settings. After trimming, we obtain on average 48.7 M (6.6 SD) trimmed RNA read pairs for each biological and technical replicate of hybrid soma and 51.1 M (5.2 SD) for each biological and technical replicate of the reproductive tract. In order to avoid mapping biases to the reference when estimating ASE, we built a custom pseudogenome using the FastaAlternateReferenceMaker walker in GATK v3.8 (McKenna et al. 2010) to hard-mask 4,670,197 SNPs between *P. citri* and *P. ficus* in the PCITRI.V0 reference. We separately mapped reads from each lane and technical replicate for all three soma and reproductive tract biological replicates against the pseudogenome using STAR v2.5.2b (Dobin et al. 2013) in the two-pass mode, marked duplicates with Picard v2.17 (http://broadinstitute.github.io/picard, last accessed March 02, 2021) and quantified expression levels using RSEM v1.3.0 (Li and Dewey 2011). Due to high consistency across technical replicates for all samples (supplementary fig. S9, Supplementary Material online), we decided to merge BAM files from both technical replicates to estimate ASE estimation for each biological replicate (113.1–157.8 M reads per sample in each merged BAM file).

We used the ASEReadCounter walker in GATK v3.7 to retrieve maternal (*P. citri*) and paternal (*P. ficus*) allele counts at discriminant SNPs with the following settings: -U ALLOW_N_CIGAR_READS -minDepth 30 --minBaseQuality 20 --minMappingQuality 255. Then, we applied a series of filters to remove low-confidence SNP positions from the ASE data set (supplementary fig. S2, Supplementary Material online). First, we only kept SNPs present in all three tissue replicates. Second, we removed SNPs where the sum of uniquely mapped reference and alternate allele counts was <90% of the total read depth at that position in at least one replicate. Third, we used the male *P. citri* transcriptomes to remove polymorphic sites within *P. citri* undetected during SNP filtering. To do so, we mapped RNA-seq reads from the three independently sequenced pools of adult *P. citri* males to the pseudogenome, passed the merged BAM file through ASEReadCounter with default settings and removed SNPs in the hybrid ASE data set where the proportion of reference alleles in pure *P. citri* males was <95%.

The same general procedure was followed to estimate ASE in intraspecific crosses, including generating pseudogenomes unique to each of the three pairs of *P. citri* lines. We aligned 85.1 (16.0 SD) million trimmed read pairs per F1 sample to the pseudogenomes and run ASEReadCounter as above, but allowing a lower minimum read depth (20×) to account for the lower number of discriminant SNPs between parental lines. Then, we applied the first (SNPs present in at least three replicates) and second above filters to the intraspecific data sets to retain only high-confidence SNPs in the F1 transcriptomes (supplementary fig. S3, Supplementary Material online). After inspecting distributions of parental counts in F1 males, we discovered that one of the CB replicates exhibited a markedly distinct ASE distribution to the others, with ∼98% SNPs showing only maternal (CP1-2) alleles (supplementary fig. S10, Supplementary Material online). This led us to conclude that the mother of this cross had been erroneously classified as a virgin and had already been fertilized by a CP1-2 male prior to isolation, so this sample was excluded from subsequent analyses.

### Gene ASE Expression Analysis

SNPs in hybrid and intraspecific transcriptomes were assigned to PCITRI.V0 genomic annotation features using bedtools v2.27.1 (Quinlan and Hall 2010) and classified as exonic, intronic, intergenic or orphan (if located on an unannotated contig). For all predicted genes, we pooled maternal and paternal read counts across all exonic SNP in each sample and estimated gene ASE as fraction of reads expressed from the maternal genome (*p_m_*). Genes covered by a single SNP were removed from subsequent analysis unless the pooled read depth across groups of biological replicates (soma and reproductive tract in hybrids; genotypes in intraspecific males) was at least 100. We followed Wang and Clark (2014) to assign genes to ASE categories: for each gene, we conducted exact binomial tests (with Bonferroni correction) separately in all replicates against the null hypothesis of Mendelian expression (*p_m_* = 0.5) and genes were considered biparental (B) when the null hypothesis was not rejected and/or 0.35 ≥ *p_m_* ≤ 0.65. Significant genes were classified as exclusively maternal (M) if *p_m_* ≥ 0.95, maternally biased (MB) if 0.65 > *p_m_* < 0.95, paternally biased (PB) if 0.05 > *p_m_* < 0.35 and exclusively paternal (P) if *p_m_* ≤ 0.05.

Additionally, we performed a *G*-test of independence with Bonferroni correction for each gene to test whether ASE was homogenous across all groups of replicates (Wang et al. 2016). Genes that did not show significant heterogeneity across soma and reproductive tract replicates (in hybrids) and genotype replicates (in intraspecific males) were immediately validated. Significantly heterogeneous genes were included in the final analysis only if all replicates agreed on ASE category and significance of exact binomial test. After removing the genes that failed to meet these criteria, we estimated a combined *p_m_* at gene by pooling paternal and maternal SNP counts from all groups of replicates and performing a final exact binomial test for each gene with these pooled counts.

Finally, we discarded low-expressed genes (TPM < 1 on average across replicates) and, for intraspecific crosses, we only kept genes present in both cross directions for each of the three combinations of parental lines. We incorporated reciprocal information in the intraspecific crosses by averaging *p_m_* between each pair of reciprocal genotypes and classifying them into ASE categories using the *p_m_* thresholds described above. Genes that differed in category of ASE between reciprocal genotypes and showed a reciprocal *p_m_* difference higher than the average across validated genes for each pair were not assigned a final ASE category.

### Functional Annotation of Genes

We performed a gene ontology (GO) enrichment analysis using the Fisher exact test with Benjamini–Hochberg FDR to identify functional categories enriched in all annotated biparentally and paternally biased genes in soma and reproductive tract of hybrid males and in whole intraspecific F1 males using GOATOOLS (Klopfenstein et al. 2018). For intraspecific crosses, we tested genes that had been classified as biparental in at least one of the crosses. To reduce bias of enrichment analysis (Timmons et al. 2015), the background gene population was restricted to the gene data set with ASE information for each group.

Biparentally, paternally biased and paternal-only genes were further investigated. In addition to a default blastp search against UniProtKB/Swiss-Prot with an *e*-value cutoff of 1*e*^−10^, we identified reciprocal orthologues in the proteomes of *D. melanogaster* and *Acyrthosiphon pisum* using BLASTp v2.7.1+ (*E*-value ≤ 1e^−25^) and a modified version of the rbbh.py script (https://github.com/DRL, last accessed March 02, 2021).

### Calculating Chromosomal Distribution, Sex-Specificity and Rates of Molecular Divergence of Genes with Parent-of-Origin Information

The recent publication of a chromosome level assembly for another mealybug species, *P. solenopsis* (Li et al. 2020), created an opportunity to investigate chromosomal biases in parent-of-origin expression in *P. citri*. Both species share a karyotype (*n* = 5), which could suggest that genome architecture is relatively conserved between these insects. We performed sequence-homology based synteny alignments using satsuma v3.1 (Grabherr et al. 2010) between our PCITRI.V0 assembly and the more complete *P. solenopsis* assembly as the target. The majority of *P. citri* scaffolds aligned to one of the five *P. solenopsis* chromosomes without ambiguity. In the rare cases that a scaffold had multiple alignments, it was assigned to the chromosome for which it had the most alignments, or (in the case of a tie between two or more chromosomes) it was left unassigned. Under these criteria, 85% of PCITRI.v0 scaffolds were assigned to a chromosome.

In order to examine sex-specificity of gene expression, we calculated the *P. citri* SPM, a proportion of expression ranging from 0 to 1 in a focal set of tissues (Xiao et al. 2010; Kryuchkova-Mostacci and Robinson-Rechavi 2017). In our case, the contrasts were male and female tissue, such that an SPM of 1 for females indicates 100% of gene expression in females, 0.5 is unbiased expression between the sexes, and 0 is 100% expression in males. This metric is less common than differential expression analyses, which conventionally uses a 1.5-fold difference in expression (which corresponds to a 0.7:0.3 SPM) to define sex-biased genes. However, this fold-difference analysis belies how truly distinct male and female gene-expression are, with most genes expressed uniquely in one sex or the other (supplementary fig. S7, Supplementary Material online).

To estimate rates of molecular evolution for the different classes of expressed alleles, we started with the set of interspecific SNPs between *P. citri* and *P. ficus* described above. Next, we created a custom database for *P. citri* in the program SnpEff (Cingolani et al. 2012) in order to annotate variants as synonymous or nonsynonymous. To properly calculate the scaled divergence rate (d*N*/d*S*, formally defined as the number of nonsynonymous changes per nonsynonymous site divided by the number of synonymous changes per synonymous site), we required information about the number of synonymous and nonsynonymous sites in the exons of each gene. We took the set of annotated genes (.gtf file) and the reference genome (.fasta) and used a series of custom R scripts to annotate the codon-degeneracy of each protein-coding nucleotide in the genome and summed these values per-gene. With these d*N*/d*S* values, we compared evolutionary rates across expression categories for which we had consistent results across replicates (maternal-only, maternally biased and biparentally expressed genes) using a nonparametric Kruskal–Wallis test to examine whether or not there was an effect of expression type on evolutionary rate. Finding a significant result, we investigated which differences drove this pattern with a post hoc Nemenyi test. These statistical tests were all completed in R.

### Microscopy

Second, third, fourth instar and adult *P. citri* males were dissected in a drop of phosphate buffered saline (PBS) on a microscope slide using a Leica dissecting microscope. Whole reproductive tracts and Malpighian tubes were isolated from all individuals, and images of the reproductive tract were taken using a Leica S8 APO dissection microscope. Excess PBS was removed using a cotton bud and tissues were fixed directly on the slide in a drop of PFA: acetic acid (1% PFA, 45% acetic acid, 54% dH_2_O) for 5 min. Excess PFA was removed using a cotton bud and 25 µl of Vectashield Antifade Mounting Medium with Dapi (Vector Laboratories) was added directly to the tissue and cover slips applied. Slides were sealed with nail polish and stored in the dark at 4 °C. Fluorescent Z-stack images of all tissue samples were taken using a Leica TCS SPE-5 confocal microscope and then processed and merged using ImageJ (https://imagej.nih.gov/ij, last accessed March 02, 2021).

## Supplementary Material

Supplementary data are available at *Molecular Biology and Evolution* online.

## Data Availability

All RNA-seq and DNA-seq data are available at the European Nucleotide Archive under study accession number PRJEB39446. The PCITRI.V0 reference genome is publicly available at https://ensembl.mealybug.org/ (last accessed March 02, 2021). Wet lab protocols and R scripts are available at github.com/agdelafilia/ASE_in_mealybugs (last accessed March 02, 2021).

## Supplementary Material

msab052_Supplementary_Data
