# Supplementary material for: Males That Silence Their Father’s Genes: Genomic Imprinting of a Complete Haploid Genome
Source: Mol Biol Evol. 2021 Mar 4;38(6):2566–81. doi: 10.1093/molbev/msab052 (PMC8136510; doi:10.1093/molbev/msab052)
Supplement: msab052_Supplementary_Data [file msab052_supplementary_data.zip › MBE-20-0751_Supplementary_Figures.pdf]

Supplementary Figures and Tables

Males that silence their father's genes: genomic imprinting of a complete haploid genome

Andrés G. de la Filia, Andrew J. Mongue, Jennifer Dorrens, Hannah Lemon, Dominik R. Laetsch, & Laura Ross

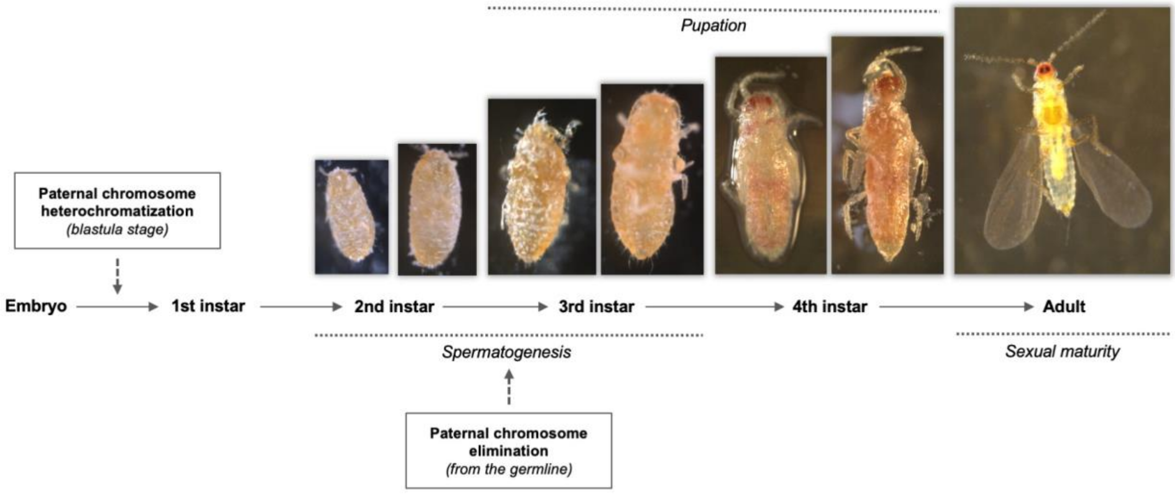

Fig. S1. Life cycle of male mealybugs.

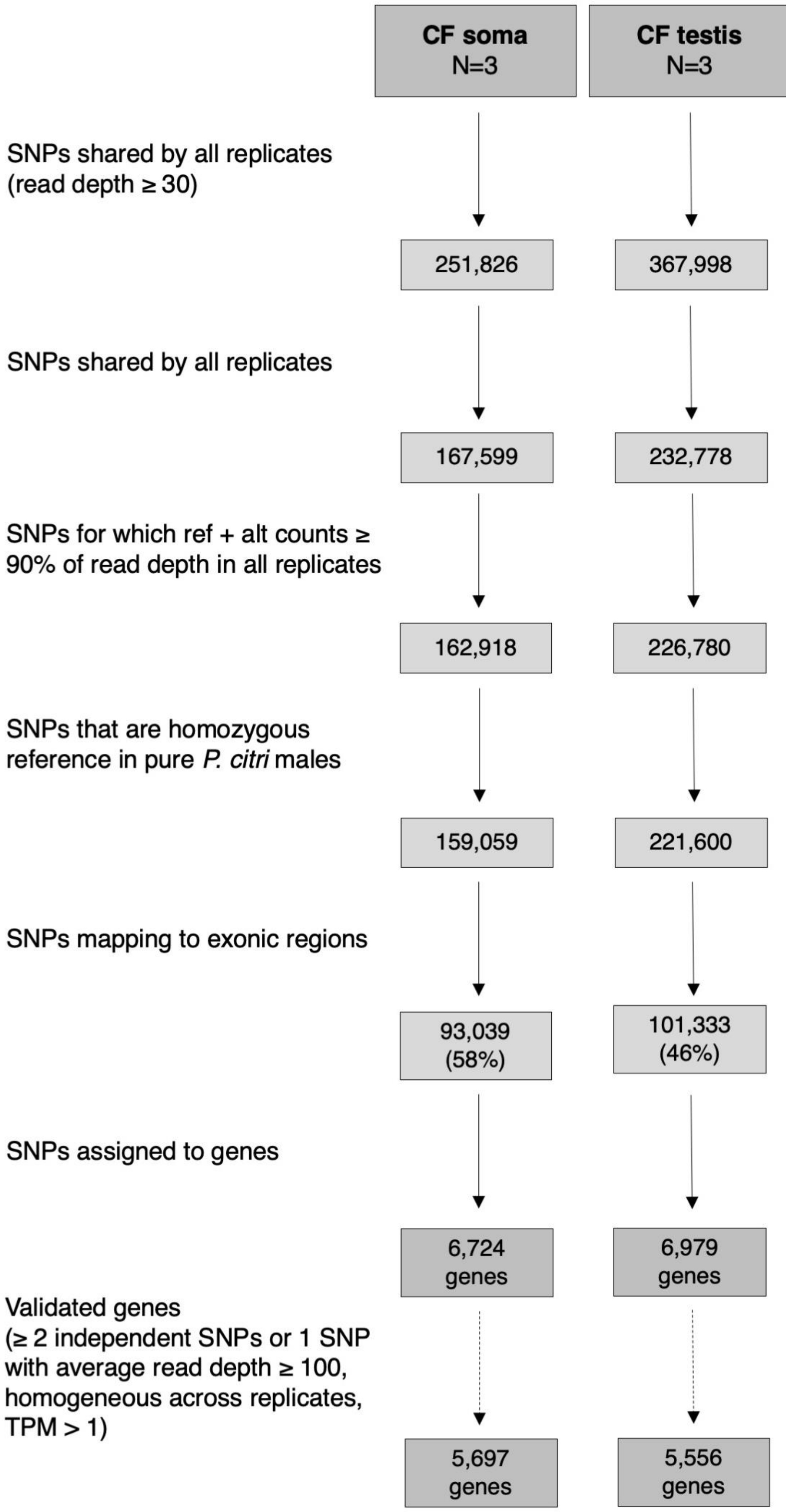
CF soma N=3
CF testis N=3
SNPs shared by all replicates (read depth ≥ 30)
251,826
367,998
SNPs shared by all replicates
167,599
232,778
SNPs for which ref + alt counts ≥ 90% of read depth in all replicates
162,918
226,780
SNPs that are homozygous reference in pure *P. citri* males
159,059
221,600
SNPs mapping to exonic regions
93,039 (58%)
101,333 (46%)
SNPs assigned to genes
6,724 genes
6,979 genes
Validated genes (≥ 2 independent SNPs or 1 SNP with average read depth ≥ 100, homogeneous across replicates, TPM > 1)
5,697 genes
5,556 genes

Fig. S2. Workflow for informative SNP filtering and assignment to genes in transcriptomes of CF hybrid males.

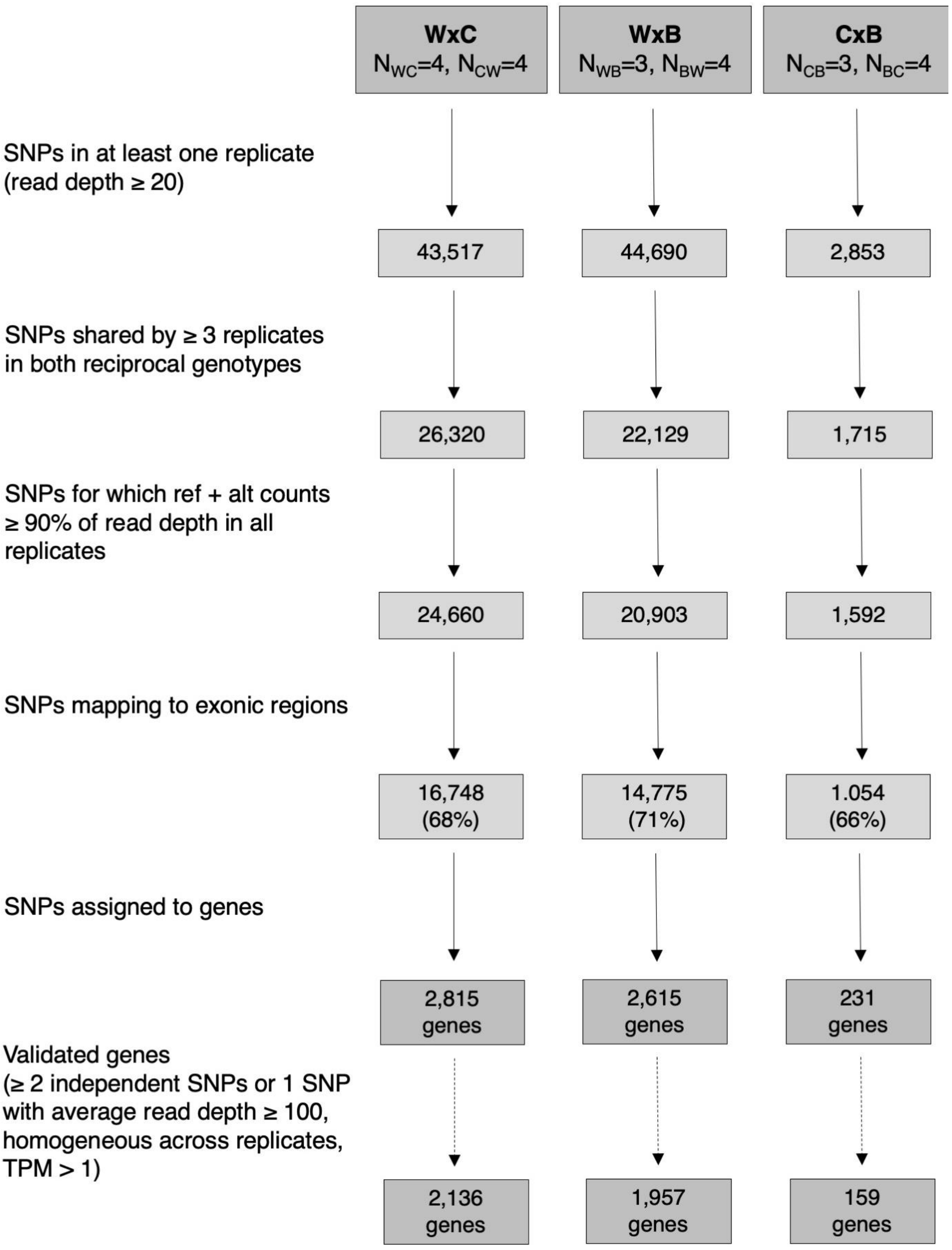

Fig. S3. Workflow for informative SNP filtering and assignment to genes in transcriptomes of intraspecific *P. citri* males

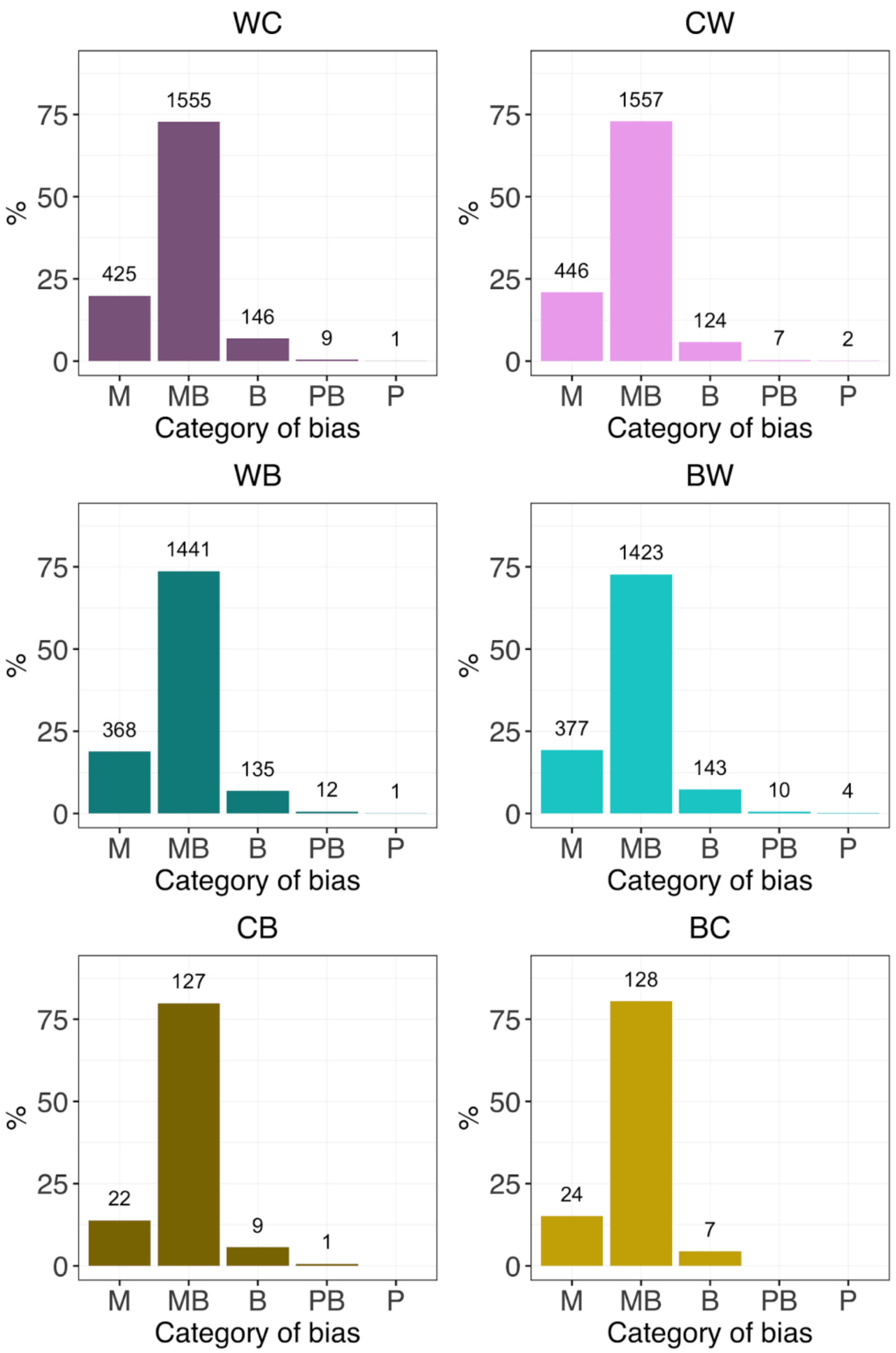

Fig. S4. Counts of genes with allele-specific information in individual intraspecific F1 genotypes according to ASE category (from completely maternal, M, to completely paternal, P)

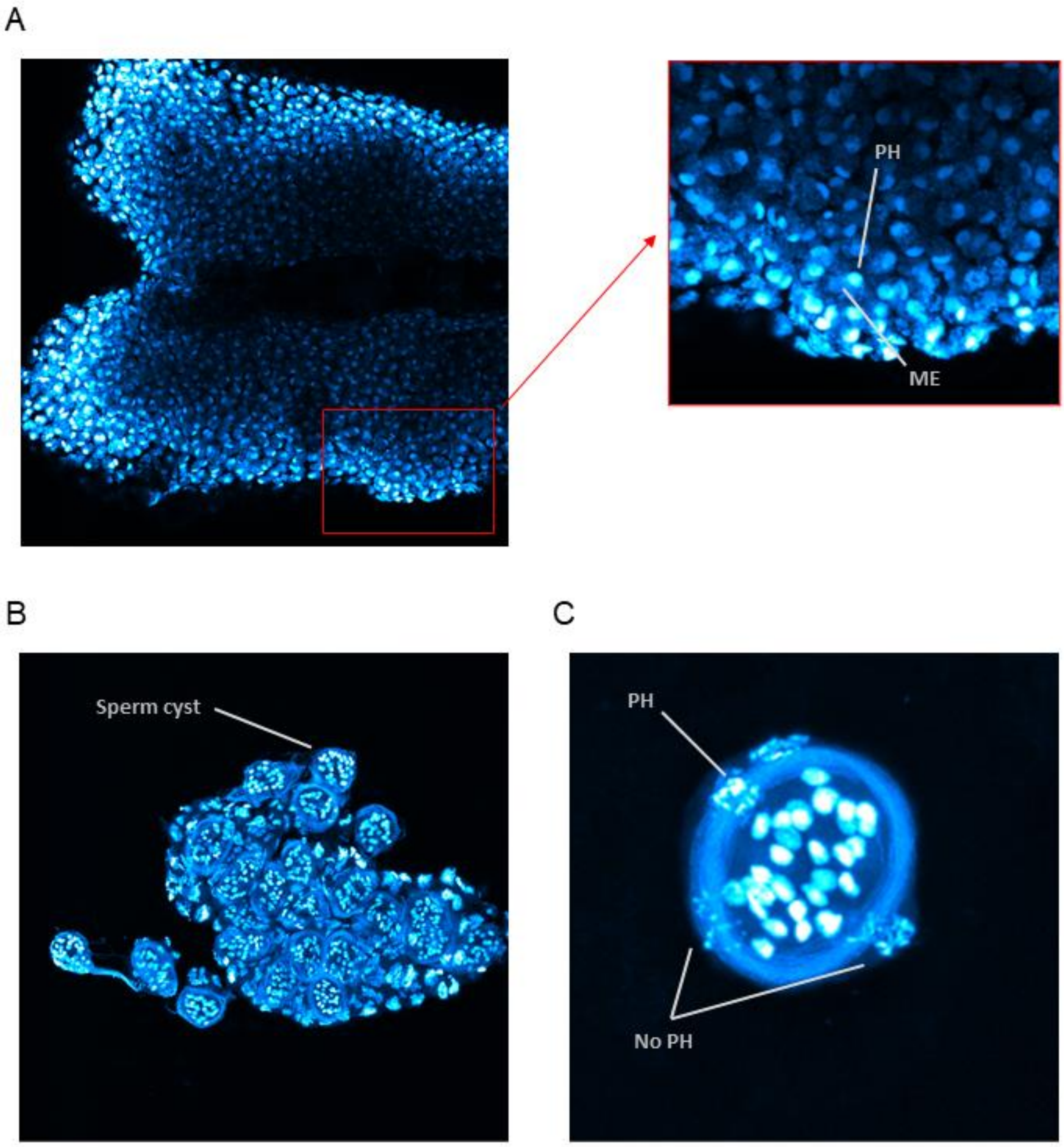

Fig. S5. Paternal genome heterochromatinisation in testes proper of 2nd/3rd instar males. (A) Early 2nd instar testis arms. All cell nuclei show heterochromatinisation of the paternal genome (PH); the maternal genome is euchromatised (ME). (B) 3rd instar male sperm cysts. Testes are organised into cysts originated from spermatogonial precursor cells. Sperm cysts are surrounded by support cells located on the cyst walls. (C) Sperm cyst from a 3rd instar male. Heterochromatinisation of the paternal genome is lost in many, but not all, of cyst wall cells.

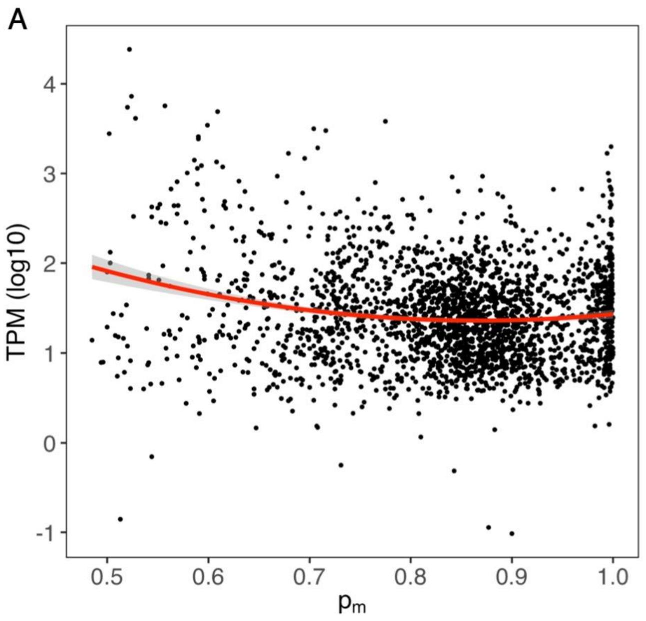

Term	Estimate	SE	T	P value
Intercept	4.4287	0.4347	10.189	< 0.0001
p_m	-7.0715	1.0937	-6.465	< 0.0001
p_m^2	4.0751	5.990	5.990	< 0.0001

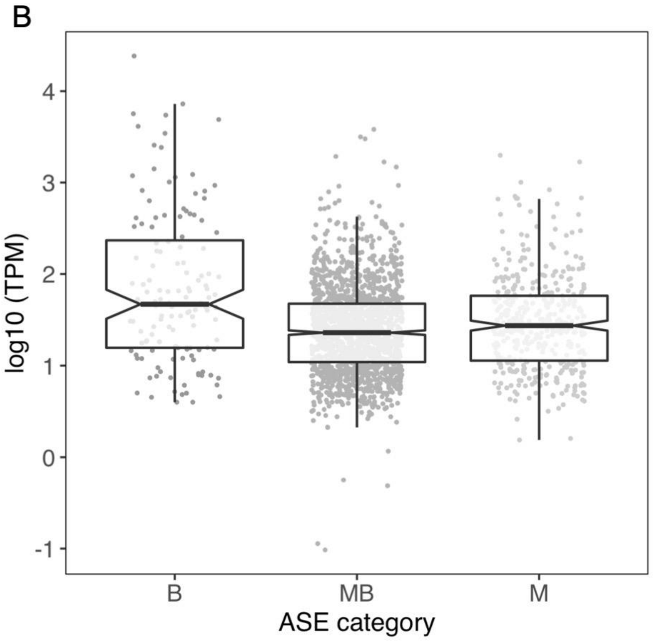

Fig. S6. (A) Results of the linear model to evaluate the relationship between gene expression levels (TPM) and bias to the maternal genome (n = 2,379 genes with ASE in intraspecific males). (B) Boxplot of TPM values across bias categories (excluding “no POE bias” genes)

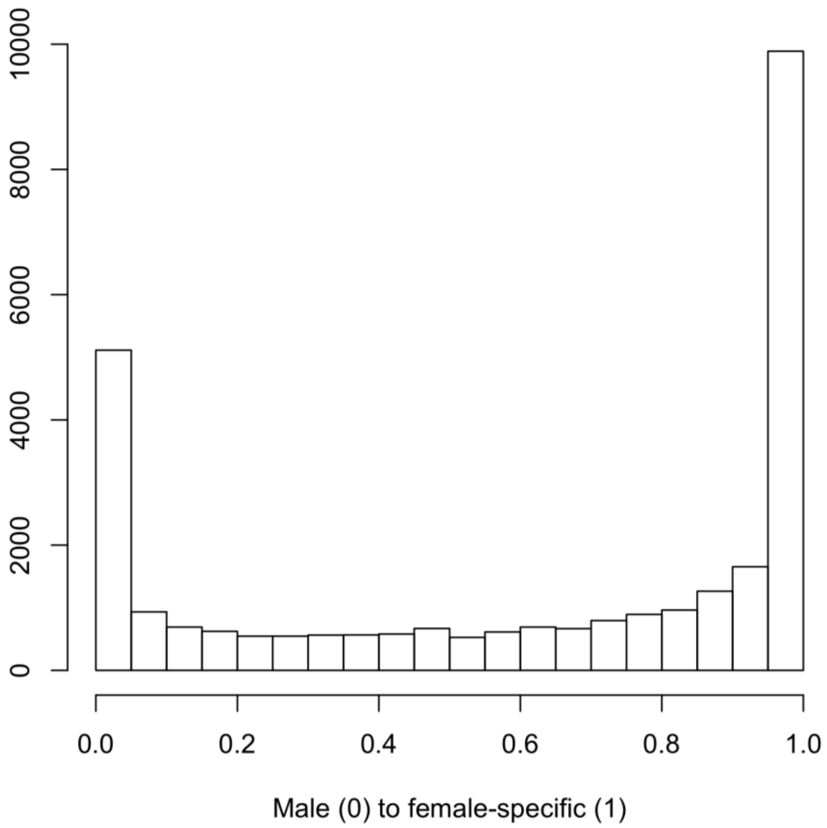

Fig. S7. Distribution of sex-specific expression, SPM, in *P. citri*

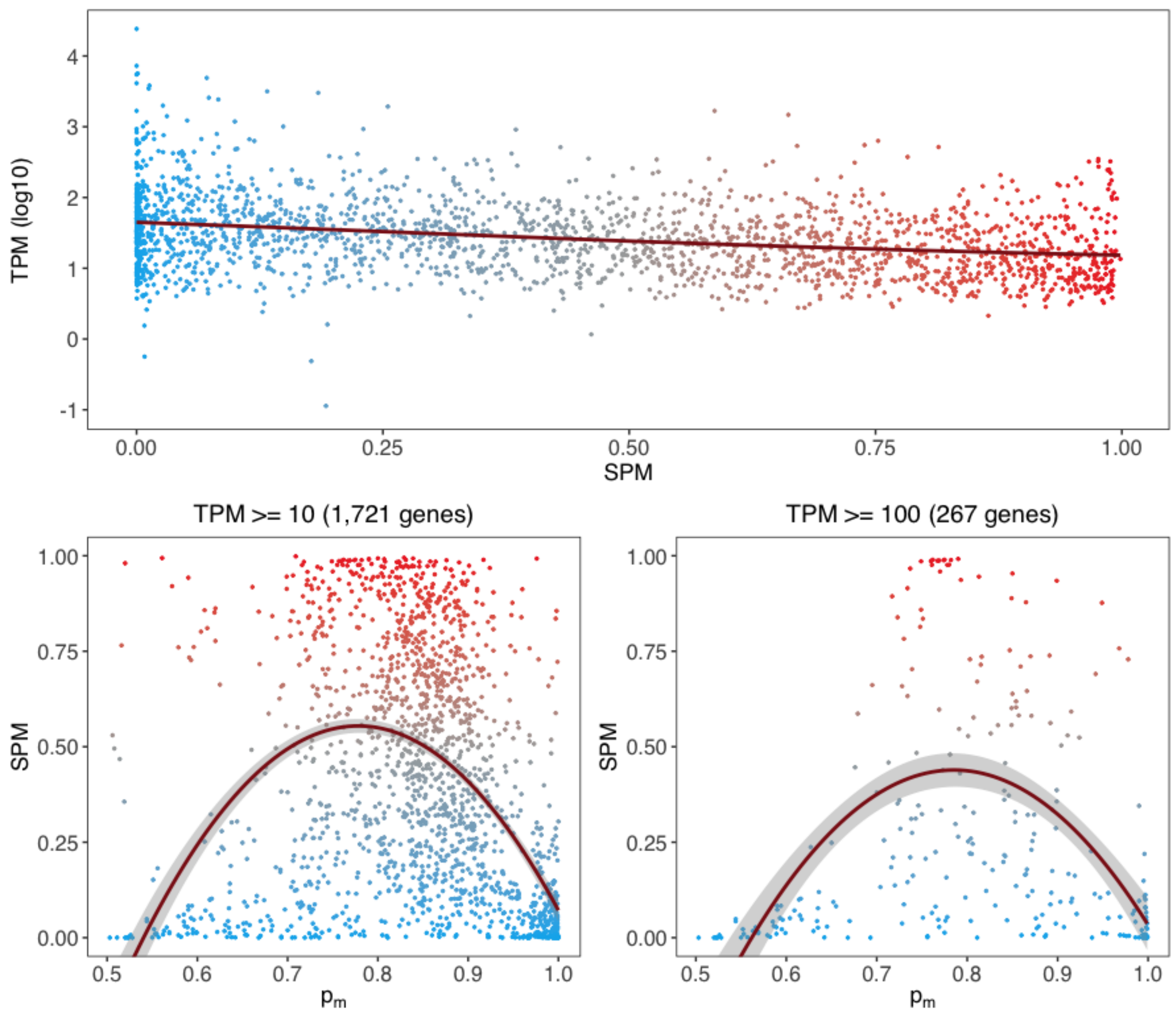

Fig. S8. (A) Relationship between expression levels (TPM) and sex-specificity of gene expression (SPM, see Fig. S7) in *P. citri* males for all 2,197 genes with consistent POE patterns. As expected, more female-specific genes tend to have lower expression levels than more male-specific genes. However, many genes with extreme female-biased expression (SPM = 0.9-0.99) show moderate expression levels in males, indicating that the high SPM is driven by increased expression in females rather than very low expression in males. (B and C) Scatterplot of SPM and bias to the maternal genome p_m after removing genes with low expression levels (B) and medium expression levels (C).

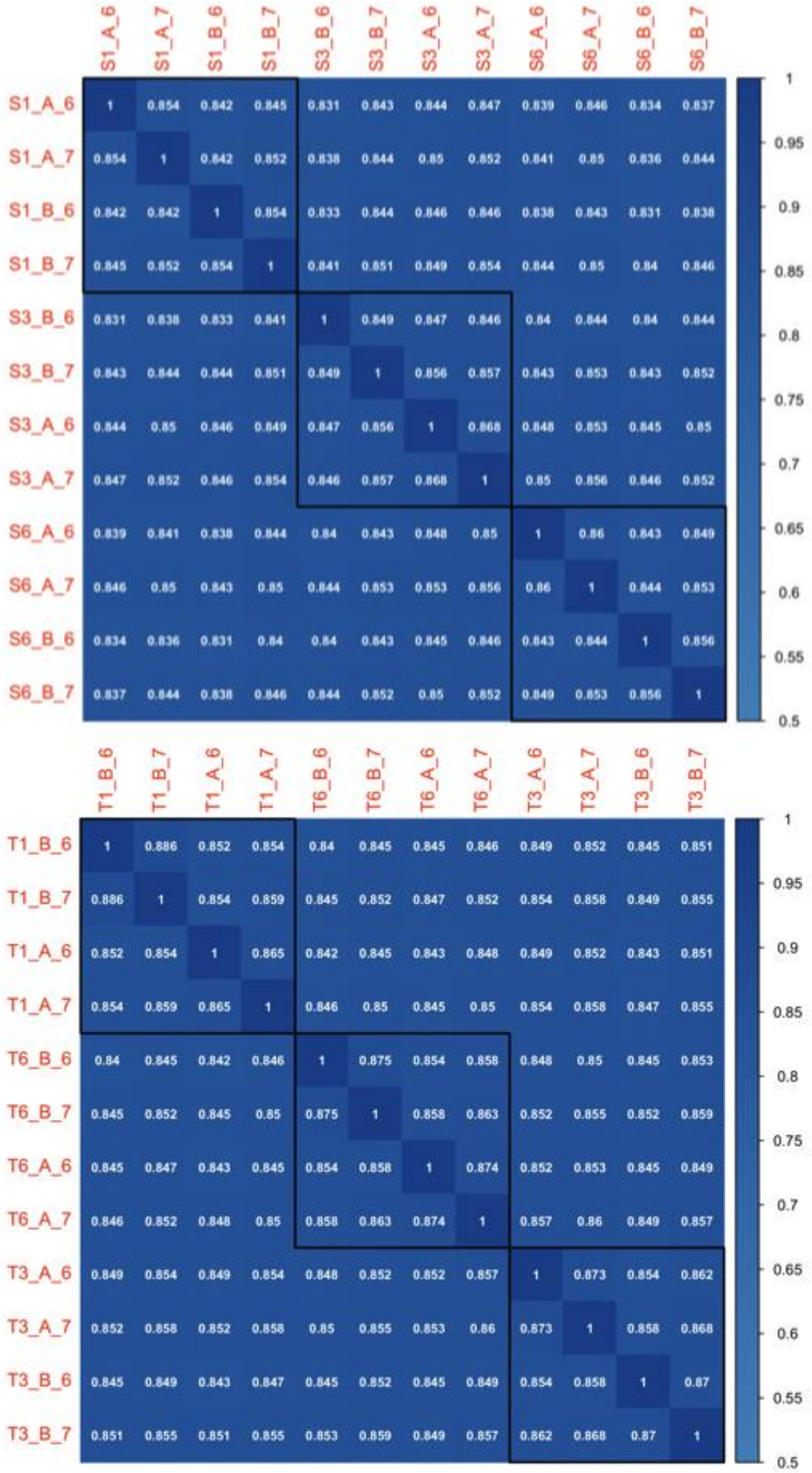

Fig. S9. Correlation matrix heatmaps of gene expression levels (TPM) between samples. Two independent cDNA amplifications (A, B) were performed for each biological replicate of hybrid

soma (S1, S3, S6) and hybrid reproductive tract (T1, T3, T6). For each tissue, 6 TruSeq Nano libraries were sequenced on two HiSeq 4000 lanes (6, 7). To assess consistency between lane and technical replicates, we mapped each of the 12 soma and reproductive tract RNA-seq datasets separately to the pseudogenome and estimated gene expression values as TPM. The heatmaps show correlation coefficients (Spearman's ρ) across the soma (top panel) and reproductive tract (bottom panel) RNA-seq datasets. We used the "hclust" and "addrect" options in the corrplot R package (https://github.com/taiyun/corrplot) to order samples by hierarchical clustering and identify the three main clusters, which correspond to the biological replicates in both hybrid soma and reproductive tract.

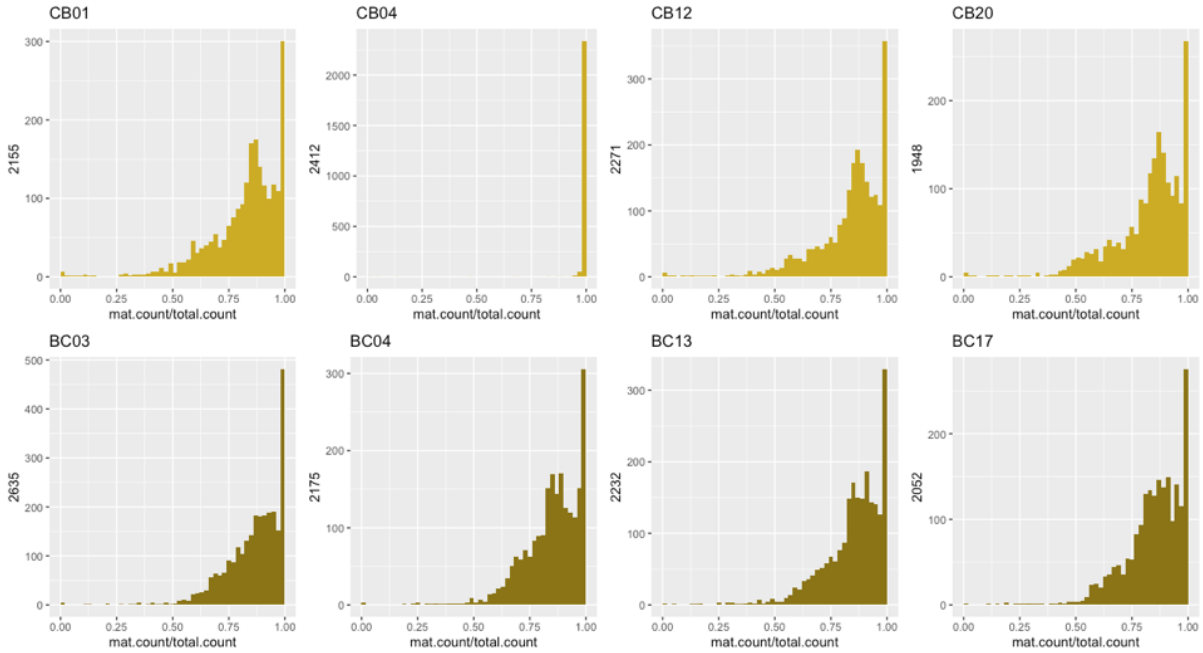

Fig. S10. Histograms of expression biases to maternal genome, p_m, at SNP level in all F1 samples from crosses between CP1-2 and BGOX-6 parents (CB, CP1-2 mothers; BC, BC, BGOX-6 parents). CB04 was excluded from the analysis.

Table S1. Counts and average bias to the maternal genome p_m (SD) of validated informative SNPs in CF transcriptomes, grouped by annotation feature.

CF soma	**Annotation**	**N SNPs**	**p_m**
	Exonic	93039 (58.5%)	0.86 (0.13)
	Intergenic	46544 (29.3%)	0.92 (0.11)
	Intronic	18401 (11.4%)	0.91 (0.13)
	Orphan	1075 (0.7%)	0.93 (0.10)
CF reproductive tract	**Annotation**	**N SNPs**	**p_m**
	Exonic	101333 (45.7%)	0.96 (0.07)
	Intergenic	81002 (36.6%)	0.98 (0.06)
	Intronic	36403 (16.4%)	0.98 (0.05)
	Orphan	2862 (1.3%)	0.98 (0.08)

Table S2. Counts and average biases to the maternal genome p_m (SD) within reciprocal genotypes of validated informative SNPs in intraspecific F1 transcriptomes, grouped by annotation feature.

W x C	**Annotation**	**N SNPs**	**p_m (WC)**	**p_m (CW)**
	Exonic	16748 (67.9%)	0.84 (0.13)	0.85 (0.13)
	Intergenic	6871 (27.9%)	0.84 (0.15)	0.86 (0.14)
	Intronic	932 (3.8%)	0.78 (0.22)	0.82 (0.18)
	Orphan	109 (0.4%)	0.85 (0.14)	0.84 (0.17)
W x B	**Annotation**	**N SNPs**	**p_m (WB)**	**p_m (BW)**
	Exonic	14775 (70.7%)	0.84 (0.13)	0.83 (0.15)
	Intergenic	5414 (25.9%)	0.84 (0.14)	0.84 (0.16)
	Intronic	603 (2.9%)	0.80 (0.20)	0.81 (0.19)
	Orphan	111 (0.5%)	0.90 (0.12)	0.87 (0.16)
C x B	**Annotation**	**N SNPs**	**p_m (CB)**	**p_m (BC)**
	Exonic	1054 (66.2%)	0.81 (0.13)	0.83 (0.11)
	Intergenic	438 (27.5%)	0.82 (0.14)	0.82 (0.15)
	Intronic	53 (3.3%)	0.84 (0.11)	0.84 (0.12)
	Orphan	111 (3.0%)	0.95 (0.11)	0.96 (0.05)

Table S3. GO enrichment analysis of 172 genes with biparental (B) or predominantly paternal (PB, P) expression in soma of CF hybrid males against a background population of 3,193 genes with allele-specific information and associated GO terms. All significant GO terms are enriched.

GO term	***Domain***	***Ratio***	***Ratio in pop***	***FDR***	***Gene ID***
GO:0006412 (translation)	BP	17/ 172	59/ 3193	7.7e-5	g12633, g13997, g1425, g19442, g25371, g26288, g34399, g37033, g38122, g38200, g38206, g38423, g5843, g636, g674, g762, g9816
GO:0055114 (oxidation-reduction process)	BP	21/ 172	171/ 3193	4.7e-2	g10992, g13384, g14250, g14596, g17372, g23111, g2512, g28606, g33094, g34137, g34342, g3639, g36400, g37858, g38672, g39758, g4370, g6152, g7114, g8627, g8706
GO:0005840 (ribosome)	CC	17/ 172	58/ 3193	2.1e-5	g12633, g13997, g1425, g19442, g25371, g26288, g34399, g37033, g38122, g38200, g38206, g38423, g5843, g636, g674, g762, g9816
GO:0003735 (structural constituent of ribosome)	MF	17/ 172	60/ 3193	1.3e-4	g12633, g13997, g1425, g19442, g25371, g26288, g34399, g37033, g38122, g38200, g38206, g38423, g5843, g636, g674, g762, g9816
